# Blue carbon of Mexico, carbon stocks and fluxes: a systematic review

**DOI:** 10.7717/peerj.8790

**Published:** 2020-04-06

**Authors:** Jorge A. Herrera-Silveira, Monica A. Pech-Cardenas, Sara M. Morales-Ojeda, Siuling Cinco-Castro, Andrea Camacho-Rico, Juan P. Caamal Sosa, Juan E. Mendoza-Martinez, Eunice Y. Pech-Poot, Jorge Montero, Claudia Teutli-Hernandez

**Affiliations:** 1 Departamento Recursos del Mar, Centro de Investigación y de Estudios Avanzados (CINVESTAV) del Instituto Politécnico Nacional Unidad Mérida, Mérida, Yucatán, México; 2 Laboratorio de Ecología, Unidad Multidisciplinaria de Docencia e Investigación de la Facultad de Ciencias, Unidad Sisal, Universidad Nacional Autónoma de México, Mérida, Yucatán, México

**Keywords:** Blue carbon, Mangroves, Seagrasses, Carbon stocks, Climate change

## Abstract

Mexico has more than 750,000 ha of mangroves and more than 400,000 ha of seagrasses. However, approximately 200,000 ha of mangroves and an unknown area of seagrass have been lost due to coastal development associated with urban, industrial and tourist purposes. In 2018, the approved reforms to the General Law on Climate Change (LGCC) aligned the Mexican law with the international objectives established in the 2nd Article of the Paris Agreement. This action proves Mexico’s commitment to contributing to the global target of stabilizing the greenhouse gas emissions concentration in the planet. Thus, restoring and conserving mangrove and seagrass habitats could contribute to fulfilling this commitment. Therefore, as a first step in establishing a mitigation and adaptation plan against climate change with respect to conservation and restoration actions of these ecosystems, we evaluated Mexican blue carbon ecosystems through a systematic review of the carbon stock using the standardized method of Preferred Reporting Items for Systematic Reviews and Meta-Analyses (PRISMA). We used the data from 126 eligible studies for both ecosystems (*n* = 1220). The results indicated that information is missing at the regional level. However, the average above and below ground organic carbon stocks from mangroves in Mexico is 113.6 ± 5.5 (95% CI [99.3–118.4]) Mg C_org_ ha^−1^ and 385.1 ± 22 (95% CI [344.5–431.9]) Mg C_org_ ha^−1^, respectively. The variability in the C_org_ stocks for both blue carbon ecosystems in Mexico is related to variations in climate, hydrology and geomorphology observed along the country’s coasts in addition to the size and number of plots evaluated with respect to the spatial cover. The highest values for mangroves were related to humid climate conditions, although in the case of seagrasses, they were related to low levels of hydrodynamic stress. Based on the official extent of mangrove and seagrass area in Mexico, we estimate a total carbon stock of 237.7 Tg C_org_ from mangroves and 48.1 Tg C_org_ from seagrasses. However, mangroves and seagrasses are still being lost due to land use change despite Mexican laws meant to incorporate environmental compensation. Such losses are largely due to loopholes in the legal framework that dilute the laws’ effectiveness and thus ability to protect the ecosystem. The estimated emissions from land use change under a conservative approach in mangroves of Mexico were approximately 24 Tg CO_2_e in the last 20 years. Therefore, the incorporation of blue carbon into the carbon market as a viable source of supplemental finance for mangrove and seagrass protection is an attractive win-win opportunity.

## Introduction

Coastal ecosystems are critical for the maintenance of biodiversity and human well-being by providing diverse benefits and ecosystem services, including protection against storms and mean sea level rise, as well as the prevention of coastal erosion, water quality regulation, nutrient recycling, and provision of habitats for high diversity commercial species, among others (Gautier, Amador & Newmark, 2001; Kathiresan & Rajendran, 2005; Mazda, Kobashi & Okada, 2005; Alongi, 2008; Bouillon & Connolly, 2009; Koch et al., 2009; Wang et al., 2010; Yulianto, Soewardi & Adrianto, 2016).

Mangroves, seagrasses and salt marshes are known as blue carbon ecosystems; they sequester greenhouse gases and store more organic carbon over the long term per unit area than terrestrial forests, and they are now recognized for their role in the climate change mitigation (Pendleton et al., 2012). Despite these benefits, blue carbon ecosystems are among the most threatened ecosystems, and their relatively low coverage <0.5% (Duarte et al., 2013) is the result of natural fragility and human-induced impacts.

International groups, such as the Intergovernmental Panel on Climate Change (IPCC), have begun to recognize the climate mitigation value of these ecosystems and included them in the 2016 update to the 2003 Wetlands Supplement (IPCC, 2014). At an international level, it has been recognized that C_org_ sequestration and storage in the vegetation and soil of blue carbon ecosystems could be a key component of mitigation strategies in the face of climate change. Thus, actions that conserve, restore, and sustainability use coastal wetlands are needed to avoid emissions and maintain (and where possible enhance) coastal wetland sequestration and storage. These actions contribute to global and national carbon management and increase the resilience of the socioecological ecosystem (Wolanski et al., 2004; Saintilan et al., 2013; Sutton-Grier & Moore, 2016; Lovelock, Fourqurean & Morris, 2017; Macreadie et al., 2017).

Mexico has one of the largest extensions of blue carbon ecosystems and is among the areas with the greatest coverage in the tropical and subtropical Western Hemisphere. The Mexican Federal Government reports 755,555 ha of mangrove and 461,059 ha of seagrasses (Valderrama-Landeros et al., 2017; CONABIO, 2018). However, estimations of their extensions have varied over time according to the precision of the methods (aerial photos, satellite images, number of sites verified “in situ”), and special attention is required for seagrasses due to the scarce reports on their extension, which differ significantly (Table 1). For mangroves, Mexico’s main species are *Rhizophora mangle*, *Avicennia germinans* and *Laguncularia racemosa* (Valderrama-Landeros et al., 2017); and for seagrasses, the main species are *Halodule wrightii*, *Syringodium filiforme*, *Thalassia testudinum* and *Zostera marina* (CONABIO, 2018).

**Table 1:** Reported extension for Mexican mangroves and seagrasses in the last 48 years (1970–2018).

Period (decade)	Mangroves extension (ha)	Seagrass extension (ha)	References
1970–1980	1,420,000^1^ 1,124,000^2^ 856,405^3^	–	1, 2, 3
1981–1990	985,600^2^ 855,566^3^	–	2,3
1991–2000	932,800^4^ 885,000^2^	–	2, 4
2001–2010	820,000^2^ 774,134^3^ 773,854^3^ 764,774^3^	688,230^7^	2, 3, 7
2011–2018	741,917^6^ 939,521^8^ 775,555^3^	919,300^10^ 456,059–461,058^11^	3, 6, 8, 10, 11

**Notes.**

Data from: ^1^Flores et al. (1971); ^2^FAO (2007); ^3^Valderrama-Landeros et al. (2017); ^4^Spalding, Blasco & Field (1997); ^6^Giri et al. (2011); ^8^INEGI (2014); ^7^Green & Short (2003); ^10^CCA (2016); ^11^CONABIO (2018).

**Figure 1:**
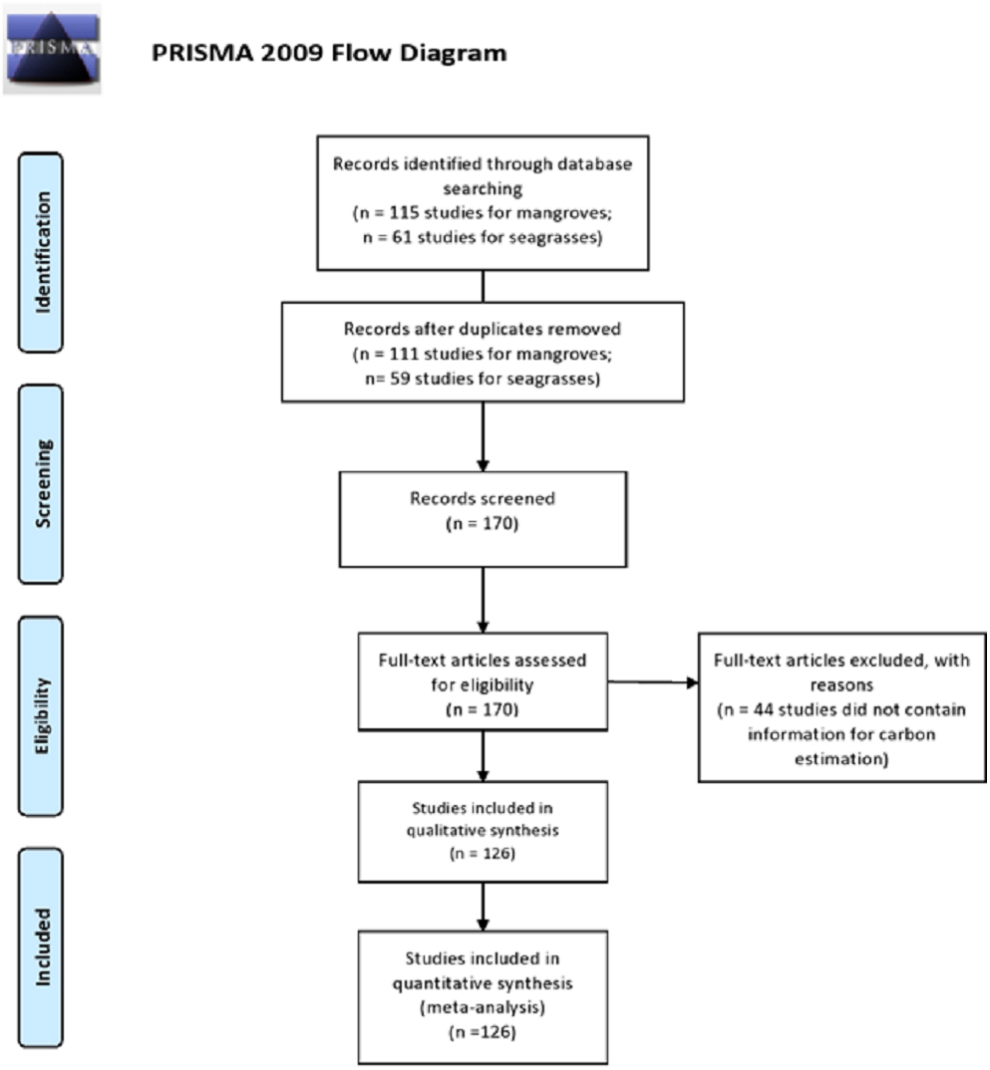
PRISMA flow diagram of the literature selection process for the systematic review of Mexican blue carbon stocks and fluxes. From: Moher et al. (2009b).

Recently, academia, nongovernmental organizations and governmental groups have created synergies to increase scientific knowledge concerning blue carbon ecosystems, and the C_org_ reserves of several ecosystems have been quantified and mapped (Caamal-Sosa et al., 2011; Adame et al., 2013; Ramírez-Ramírez, Medina-Gómez & Herrera-Silveira, 2015; Adame & Fry, 2016; Ezcurra et al., 2016; Kauffman et al., 2016; Medina-Gómez et al., 2016; Cinco-Castro et al., 2017; Ochoa-Gómez et al., 2019). In this systematic review, we checked more than 150 reports with data related to blue carbon stocks and fluxes in Mexico (Fig. 1).

The storage and fluxes of C_org_ in blue carbon ecosystems mainly depend on the community structural characteristics and extensions, which are the result of the addition of particularities of climate, geomorphology, hydrology and human land use. In Mexico, there is a natural and human-induced heterogeneity along the 11,592 km (De la Lanza Espino, Pérez & Pérez, 2013) of coast, resulting in a mosaic of spatial and structural arrangements of mangrove and seagrass communities. Traditionally, management schemes do not consider all the abovementioned criteria.

In the case of seagrasses, both the bathymetry gradient and coastal current velocities influence the water transparency, which is one of the key variables for seagrass development. The variability in water transparency is a factor related to the morphometric characteristics of the plants, which determine the above ground C_org_ assessments of this ecosystem (Fourqurean et al., 2012a). However, Mexico has insufficient information on seagrasses at the high spatial resolution required for use as a reference criterion for seagrass zonation. Currently, the Mexican government is requesting information from mangrove and seagrass C_org_ stocks under different criteria that allow for the development of conservation and restoration policies at different scales (INECC, 2018). In this context, it is necessary to consider the ecological and environmental scenarios in which mangroves and seagrasses develop to improve local, regional and national carbon inventories.

Mexico ranks 13 in the list of countries with the largest CO_2_ emissions (IEA, 2014), derived from the use and burning of fossil fuels representing 1.37% of global emissions in 2012; however, and unknown amount of CO_2_ emissions is contributed by degraded or destroyed blue carbon ecosystems, which are able to release the carbon they have stored for centuries into the atmosphere and oceans and become sources of greenhouse gases (Pendleton et al., 2012). It has been estimated by experts that as much as 1.02 billion tons of carbon dioxide are being released annually from degraded coastal ecosystems, which is equivalent to 19% of emissions from tropical deforestation globally (Pendleton et al., 2012). Changes in the coverage of Mexico’s blue carbon ecosystems over time and their relation to changes in land use have been difficult to quantify due to the lack of long-term evaluation programs.

Mexico is one of the 175 countries that have signed onto the Paris Agreement, and it has committed to “increase carbon capture and coastal protection with the implementation of conservation and recovery schemes for coastal and marine ecosystems, such as coral reefs, mangroves, seagrasses and dunes”, through Mexico’s Nationally Determined Contribution with the Adaptation category (INECC, 2018). However, insufficient data have been collected in Mexico, and no complete synthesis of the mitigation value or the greenhouse gases (GHG) emissions related to mangroves and seagrasses existed until now. This comprehensive review is a first step in providing a tool for decisionmakers to develop efficient strategies aimed at reducing carbon emissions from the loss of these ecosystems while also protecting current levels of carbon capture and storage as well the many ecosystem services provided by mangroves and seagrasses.

## Methods

A systematic review was conducted at the country level to assess the carbon stocks and fluxes of blue carbon ecosystems in Mexico. We use the Preferred Reporting Items for Systematic Reviews and Meta-Analyses (PRISMA) (Fig. 1) framework and protocols (Moher et al., 2009a).

### Information sources and search strategy

An electronic literature search was performed for carbon stocks and carbon fluxes in mangroves and seagrasses from Mexico. We use PubMed (MEDLINE) and Web of Science as the primary sources for searches and include open access publications. Google Scholar was a secondary source used to acquire additional literature (thesis, technical reports), and the inclusion of gray literature is recommended for systematic reviews to minimize publication bias (Koricheva, Gurevitch & Mengersen, 2013; Pullin & Stewart, 2006).

We also include published databases on C_org_ stocks from Herrera-Silveira et al. (2018a) and Herrera-Silveira et al. (2018b), and the Mexican Carbon Program (PMC), which have been validated and reviewed by experts and organizations of the federal government and civil society (CONABIO and CONAFOR) and subjected to public consultation (http://pmcarbono.org/pmc/). In the initial phase, titles and abstracts from network were screened to identify potential eligible studies. In the second phase, full texts of the remaining articles were read to determine if they meet the inclusion and exclusion criteria. When disagreement emerged regarding the eligibility of studies, the main author Jorge A. Herrera Silveira made the final decision.

The search of carbon stocks and fluxes extended from 1987 to 2018 and included keywords (exclusively in Spanish and English) population (“mangrove”, OR “seagrass”, OR “Submerged aquatic vegetation”, OR “wetlands”, OR “coastal basin”, OR “coast”), AND compartments (“Forest structure”, OR “ecosystem structure”, OR “DBH”, OR “biomass”, OR “above ground” AND “biomass”, OR “below ground” AND “biomass”, OR “litter productivity”, OR “carbon” AND “flux”, OR “decomposition” OR “soil” AND “carbon”, OR “soil” AND “organic matter”, OR “sediment” AND “organic matter”, OR “soil” AND “bulk density”, OR “sediment” AND “bulk density”), AND “location” (v.g. “Gulf of Mexico”, OR “Pacific”, OR “Yucatan Peninsula”) OR “Mexican Caribbean”, OR state names (v.g. “Yucatan”, OR “Campeche”, “Quintana Roo”, OR “Tabasco”, OR “Veracruz”, OR “Tamaulipas”, OR “Baja California Sur”, OR “Baja California Norte”, OR “Sinaloa”, OR “Oaxaca”, OR “Chiapas”); OR specific site names (v.g. “Laguna de Terminos”, OR “Magdalena Bay”, OR “Sian Ka’an”, OR “La Encrucijada”, OR “La Mancha”, OR “Laguna Alvarado”, OR “San Quintín Bay”, OR “Marismas Nacionales”, OR “Celestún”, OR ” Laguna Madre”, OR “Barra de Navidad”, OR “Laguna Mar Muerto”).

The searches were undertaken independently by authors with at least five years of experience in sampling, laboratory analysis, and proven experience in data analysis and redaction of technical reports on blue carbon ecosystems. The five researchers for mangrove review were the coauthors Andrea Camacho-Rico, Monica A. Pech-Cardenas, Siuling Cinco-Castro; for sediments were Eunice Y. Pech-Poot and Juan P. Caamal Sosa; and for seagrasses were Juan E. Mendoza-Mantínez and Sara M. Morales-Ojeda.

### Eligibility criteria

We include studies and datasets that report the biomass, organic carbon stock, organic matter and bulk density in sediments per unit of area and spatially referenced or flux data from mangroves and seagrasses. For mangroves, an important criterion was the plot design (including at least three replicates) to guarantee the representativeness of the data; for seagrasses, we looked for studies that based their results on quadrants or cylindrical core sampler along transects. No conference abstracts were considered to meet the inclusion criteria.

### Data extraction and analysis

Data extraction was independently performed by authors (Andrea Camacho Rico, Monica A. Pech -Cardenas, Siuling Cinco-Castro, Eunice Y. Pech-Poot, Juan P. Caamal-Sosa, Juan E. Mendoza-Martinez and Sara M. Morales Ojeda). Study-specific variables were recorded for each entry. Many properties assessed in this review were reported as contextual environmental data rather than the primary outcome for their respective studies (no replicates or dispersion measures described), which was why bias was not reported in this study. We include entries such as geographic location and region (according to Valderrama-Landeros et al., 2017), environmental characteristics including ecological type (Lugo & Snedaker, 1974), sample, compartment (above and below ground components), mangrove fluxes by litterfall biomass and/or constant decay of litter.

**Figure 2:**
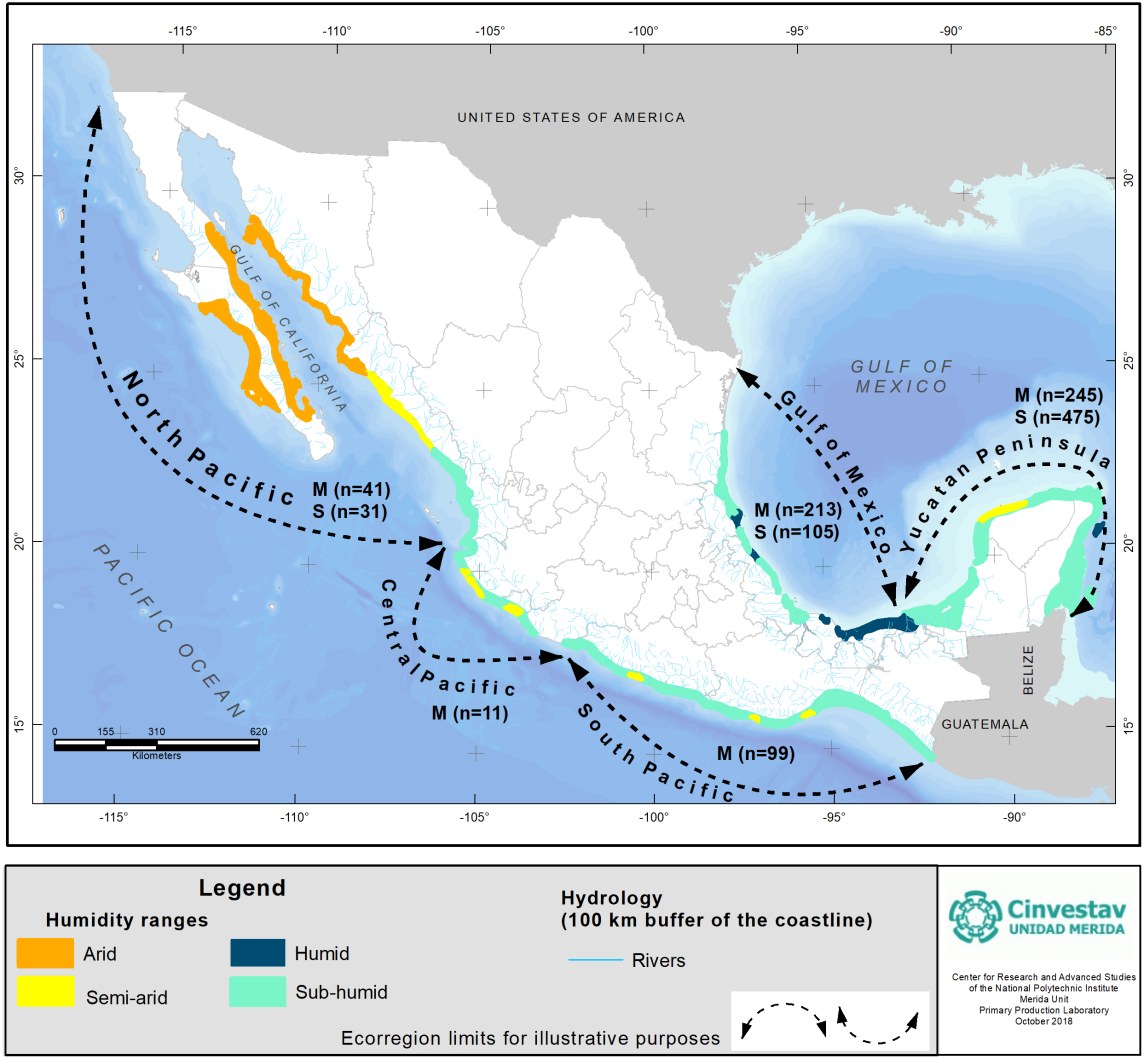
Cartographic representation of climate regions acording to humidity ranges and hydrography of Mexican coastal areas; information of data number (*n*) for mangrove (M) and seagrass (S) at each region is provided. Reference data provided by CONABIO (2009). Based on Maderey-R & Torres-Ruata, 1990 for hydrology; and García, 1990 for humidity ranges. Geographic Coordinate System, Datum WGS84.

The estimation of C_org_ stocks was carried out for mangroves by authors Monica A. Pech-Cardenas, Andrea Camacho-Rico, Juan P. Caamal-Sosa, Claudia Teutli-Hernandez and for seagrasses by Juan E. Mendoza-Martinez and Sara M. Morales Ojeda, and the supervisor was Jorge A. Herrera-Silveira. The above and below ground biomass of all the studies were converted to organic carbon using the factor 0.45 for mangroves and 0.35 for seagrasses (Fourqurean et al., 2012a; Howard et al., 2014; Kauffman & Donato, 2012). The total soil C_org_ pool was standardized to a depth of 1 m, although there are reports in mangroves of organic matter depths greater than 1 m (Caamal-Sosa et al., 2011; Adame & Fry, 2016; Ezcurra et al., 2016). The units employed to report the C_org_ stocks in the coastal blue carbon ecosystems were Mg C_org_ ha^−1^ except where indicated. For both ecosystems, our lab data were used as the control for C_org_ and for both above and below ground assessments. The georeferenced data were standardized and plotted, and corrections were made for inconsistencies in the location of the sites and derived from the different coordinate systems used in the literature. The mangrove zonation used belongs to the national official regionalization and was proposed by a panel of scientists (CONABIO, 2009 based on Lugo & Snedaker, 1974). The humidity ranges were defined according to CONABIO (2009) based on García (1988); we use humid (Am and Af variations), sub humid (Aw2, Aw1, Aw0 variations), arid (BW) and semi-arid (BS1) classifications. The ecological typology criteria used were dwarf, basin, fringe and hammock (peten) mangrove types, which could be of interest both for decision makers and for future research at regional and local scales (Fig. 2, Table 2). We report the average carbon stored per region and standard error as a measure of data dispersion

**Table 2:** Characterization of the blue carbon ecosystems in Mexico by regions.

Region	States	Climate	GT	HT	Mangrove species	Seagrasses species	Surf. Salinity	Int. Salinity	*n* Mangrove	*n* Seagrasses
North Pacific	B.C.				Ag*	**				
	B.C.S.	VA*	CL*		Rm*	*Rm*			A:13	A:22
	Son.	A	SM	2*	*Lr*	Zm	34.5 (34–37)	60.6	B:28	B:9
	Sin.	SH	RE		*Ce*					
	Nay.									
Central Pacific	Jal.				Rm*					
	Col.	SH*	RE*	ND	Lr*	ND	ND	49.7 (35–78)	A:6	ND
	Mich.		CL		*Ag*				B:5	
					*Ce*					
South Pacific	Guerr.				Ag*					
	Oax.	SH	RE*	ND	Rm*	ND	ND	32.6 (8–38)	A:69	ND
	Chiap.	H*	CL		*Lr Ce*				B:30	
Gulf of Mexico	Tam.	A			Ag*	*Hb*				
	Ver.	SH*	RE*	1*	Rm*	*Hw*	33.1 (11–38)	20.9 (3–69)	A:170	A:60
	Tab.	H	CL	2	*Lr*	*Rm*			B:43	B:45
					*Ce*	*Sf*				
						*Tt*				
Yucatan Peninsula	Camp.	A	KS*		Rm*	*Hw*				
	Yuc.	SH*	CL	1 *	Lr*	*Sf*				
	Q. Roo	H	RE	2*	*Ag*	*Tt*	37.7 (18–50)	39 (0.5-86)	A:129	A:254
					*Ce*	*Rm*			B:116	B:221

**Notes.**

Regions accoriding to CONABIO (2016). Climates are based on humidity ranges (H:Humid, SH:Sub-Humid, SA:Semi-arid, A: Arid). Geomorphological types (GT) are: Coastal lagoon (CL), Salt mash (SM), River-estuarine system (RE) and Karst system (KS). Hydrodynamic types (HT) are open (1) and closed (2). The mangrove species are: Avicennia germinans (Ag), *Rhizophora mangle* (Rm), *Laguncularia racemosa* (Lr), *Conocarpus erectus* (Ce). The Seagrass species are: *Rupia maritima* (Rm), *Zostera marina* (Zm), *Halodule beaudettei* (Hb), *Halodule wrightii* (Hw), *Syringodium filiforme* (Sf), *Thalassia testudinum* (Tt). * indicate dominance. ND, No data. n, number of observations of above ground (A) and below ground (B) C stocks.

The estimation of the GHG emission pattern due to the loss of mangrove coverage was performed based on factors recommended by the IPCC (2014) and Howard et al. (2014). The extension changes in mangroves for each region was taken from Valderrama-Landeros et al. (2017) who accomplished a national mapping using remote sensing data validated by more than 1,000 verification points and 69,000 vertical aerial photographs (Rodríguez-Zúñiga et al., 2013; Valderrama-Landeros et al., 2017). The CO_2_e emissions by mangrove loss were estimated according to the average carbon stored per region and mangrove lost area. The mangroves CO_2_e emissions were estimated by regions during 1970 and 2015 time period using a conservative approach in which a loss of 25% of the C_org_ store is assumed in response to land use change (Pendleton et al., 2012).

To present an evaluation of the uncertainty of the data in this synthesis, the author Jorge Montero analyzed the carbon stock values of mangroves and seagrasses by region and nonparametric bootstrap confidence intervals were calculated using the method of adjusted bootstrap percentile (BCa, with *B* = 10,000) and bootstrap variance estimator. The BCa values were calculate using the boot.ci function in R software and library boots (Canty & Ripley, 2019). The uncertainty was calculated using the bootstrap standard error and 95% confidence interval for z normal distribution and expressed as a percentage based on the average value.

## Results and Discussion

### Study selection

We identified 176 articles based on the search criteria; however, 50 articles were deemed inappropriate and were not used in the final analysis. Thus, a total of 1,220 data points were extracted from 59 sources for seagrasses and 67 sources for mangroves, and they were used to assess Mexican blue carbon stocks. From the first 176 articles selected, 50 were screened out, remaining 126 articles which meets the quality and requirements for this study (Fig. 1). Data on carbon fluxes were scarce, evidencing the research needs in this area. To improve the accuracy of the influence from land use on ecosystem carbon dynamics, studies which include measurements of stocks and changes along the soil profile are required (Kauffman & Bhomia, 2017). With respect to seagrasses, difficulties involved in mapping the marine environment coupled with gap information in the legislative seagrasses’ framework of Mexico have resulted in limited knowledge regarding seagrass distribution. However, the researchers and national institutions efforts in recent years entails the first official numbers for the seagrass cover in the Gulf of Mexico.

### Synthesis of mangrove stocks by above ground and below ground compartments

The average above ground tree biomass was 113.6 ± 5.2 Mg C_org_ ha^−1^, while the average below ground C_org_ (soils and roots) was 385.2 ± 22 Mg C_org_ ha^−1^, for a combined total of 498.8 Mg C_org_ ha^−1^. The below ground mean C_org_ consisted of approximately 77% of the total C_org_ stock for Mexico. This value is consistent with other reports for the C_org_ stocks in Mexico, which vary between 364 Mg C_org_ ha^−1^ and 442 Mg C_org_ ha^−1^ (Herrera-Silveira et al., 2016; Adame et al., 2018). Similar to the present work, both studies used literature and original data, suggesting that the real value of the total C_org_ stock of Mexico’s mangrove is approximately 434 Mg C_org_ ha^−1^, which is a below global average that was reported elsewhere as ranging from 885 to 937 Mg C_org_ ha^−1^ and less than Tier 1 default mangrove’s values reported by the IPCC (511 Mg C_org_ ha^−1^). Differences among reports and Tier 1 estimations are largely due to the underestimation of soil carbon stocks in global studies (Donato et al., 2011; Alongi, 2012; Hiraishi et al., 2014; Kauffman & Bhomia, 2017). In the case of this systematic review, the soil C_org_ content available in the literature only includes the first 30 cm (soil deep), and the standardized 1 m values rank from <10 to 2,233 Mg C_org_ ha^−1^ (Table 3). In general, the low values must be taken with caution as the largest C_org_ stock of those soils could be deeper than 30 cm; thus, more work on soil profiles is required according to the protocols in the cited studies for the diverse blue carbon environmental settings of Mexico.

**Table 3:** C_org_ stocks in mangroves and seagrasses by Mexican geographic regions. The values represent C_org_ average ± SE (minimun–maximun) of aboveground, belowground and total carbon per unit of area (Mg C_org_ ha -1). Uncertainty (%) for mangroves and seagrasses.

Region	Mangroves				Seagrasses			
	**AboveC**_**org**_**± S.E(Min–Max)**	**BelowC**_**org**_**± S.E(Min–Max)**	**Total AverageC**_**org**_**± S.E(Min–Max)**	**Uncertainty95% CI**	**AboveC**_**org**_**± S.E(Min–Max)**	**BelowC**_**org**_**± S.E(Min–Max)**	**Total Average C**_**org**_**± S.E(Min–Max)**	**Uncertainty95% CI**
North Pacific	58.9 ± 12 (3.8–162)	270 ± 52 (45.3–893)	204.9 ± 40 (15.5–893)	39.6	0.65 ± 0.49 (0.03–2.17)	70.9 ± 96 (0.08 -243)	26.1 ± 13 (0.031–243)	93.7
Central Pacific	117.0 ± 38 (15.1–270)	112.2 ± 0.0 (73.7–214)	210.5 ± 50 (15.1–382)	42.3	ND	ND	ND	ND
South Pacific	154.8 ± 15 (14.0–408)	663.1 ± 51 (121–1161)	397.1 ± 45 (14.1–1433)	22.2	ND	ND	ND	ND
Gulf of Mexico	152.3 ± 15 (0.6–458)	438.1 ± 76 (9.8–2003)	244.2 ± 24 (0.66–2233)	19.9	1.04 ± 1.09 (0.003–6.7)	86.8 ± 88 (0.03-299)	66.1 ± 10 (0.003–299)	64.5
Yucatan Peninsula	76.9 ± 8 (0.1–451)	353.8 ± 18 (23.7–1085)	348.9 ± 21 (4.6–1201)	12.1	0.73 ± 1.24 (0.00007–7.6)	140.8 ± 116 (0.02–757)	113.7 ± 7 (0.00007-758)	24.2

The mean downed wood stock was 15.19 ± 4.1 Mg C_org_ ha^−1^ and represented up to 12% of the C_org_ above ground reservoir. The root component represented 5–9% of the underground C_org_ stocks, with an average of 26.6 ± 2.8 Mg C_org_ ha^−1^. Fine roots should be considered as an important component of underground C_org_ sequestration due to the high productivity and decomposition rates (Adame et al., 2014; Ouyang, Lee & Connolly, 2017). Few studies have demonstrated that the below ground fine root biomass contribute significantly (>20%) to the below ground live C_org_ stock in mangrove forests (Adame et al., 2014; Robertson & Alongi, 2016; Santos et al., 2017). This component is not just important for C_org_ inventories, although their turnover rates contribute to a high C_org_ capture, which is why we consider them as a part of the subterranean carbon analysis.

### Synthesis of carbon stocks in mangroves by region and humidity range

**Figure 3:**
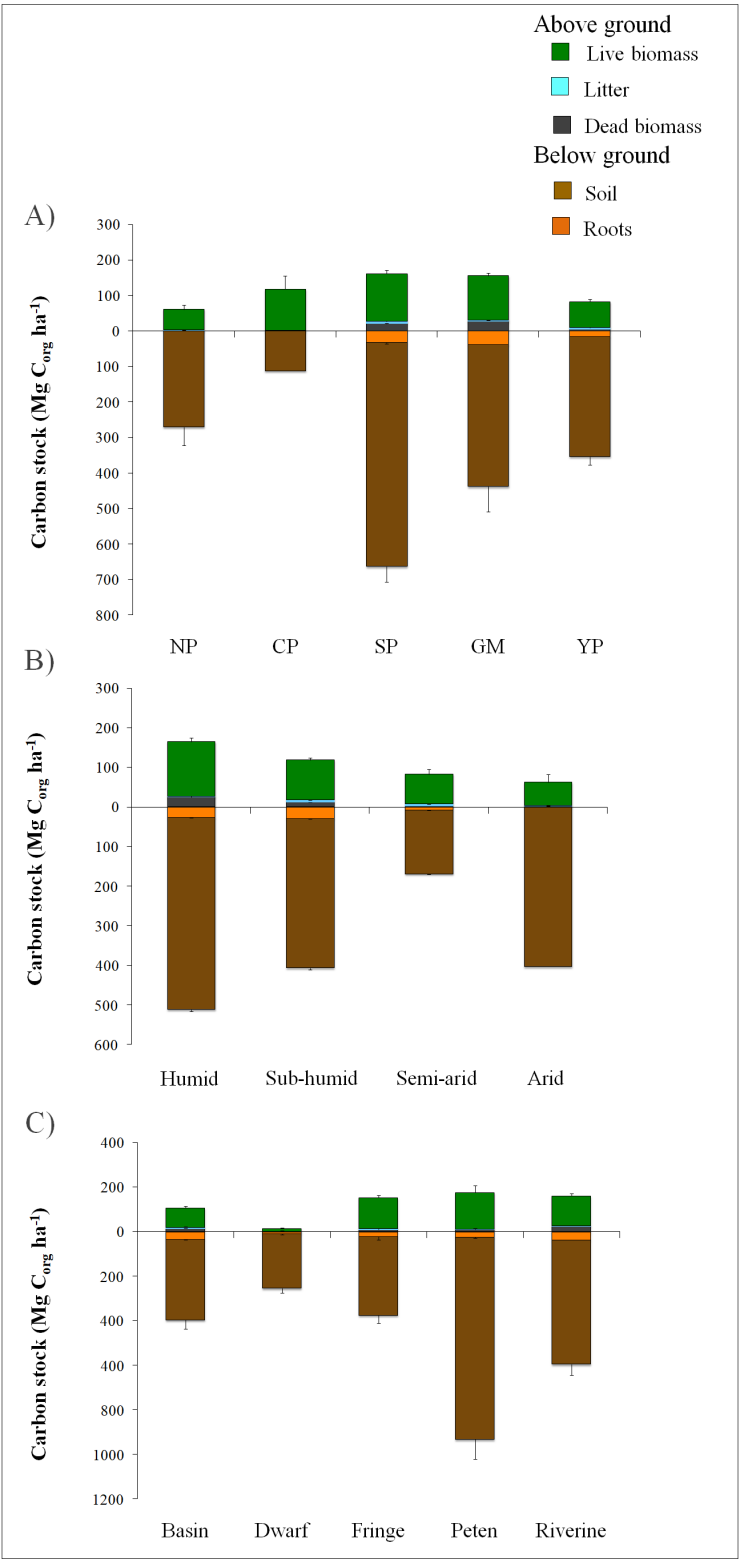
Variation in the partitioning of above and below ground contribution to the total C_org_ stocks in Mexican mangroves, according to grouping type criteria. Mexican C_org_ stocks for mangroves per unit of area in Mg ha^−1^, according to: (A) Geographic regions: North Pacific (NP), Central Pacific (CP), South Pacific (SP), Gulf of Mexico (GM) and Yucatan Peninsula (YP). (B) Climate humidity ranges. (C) Mangrove ecological type. The stocks were divided by above (litter, live and dead biomass) and below (roots and soil) ground components. Error Bars represents SE. Note the different scales used in above and below ground C_org_ stocks.

The South Pacific presents the largest above ground and below ground stock means of C_org_ (154. 8 ± 15 Mg C_org_ ha^−1^ and 663.1 ± 51 Mg C_org_ ha^−1^); the North Pacific registered the lowest stock above ground average of C_org_ (58.9 ± 12 Mg C_org_ ha^−1^), and the Central Pacific presented the lowest below ground C_org_ stock average (112.2 Mg C_org_ ha^−1^) (Fig. 3A). In the South Pacific region, the humid climate and geomorphological features create a hydrological condition that favors the development of tall mangrove forests, reaching the maximum diversity. In contrast, the Central Pacific and North Pacific coast account for a narrow continental shelf under arid or semi-arid conditions and few intertidal areas, which reduces the number of habitats available for mangroves and provides less favorable conditions for C_org_ storage. The uncertainty value recorded for the Central Pacific region (42.3%) was associated with the scarce literature registered for this region.

Meanwhile, the uncertainty of the regions of the Gulf of Mexico and the Yucatan Peninsula was low (Table 3), and the uncertainty results indicate that the database for these regions is robust for country- and global-scale analyzes.

The downed wood biomass for carbon estimation was considered only for the Gulf of Mexico, Yucatan Peninsula and South Pacific regions (Fig. 3A) due to its relevance as a source of carbon in sites exposed to hydrometeorological impacts, such as hurricanes and storms. Thus, this component is important in these regions where the frequency and intensity of these events is expected to increase in the face of climate change (Adame et al., 2013b).

The underground mangrove C_org_ storage of this synthesis varies between 48.8 and 82.1% of the total ecosystemic C_org_. The lowest values correspond to the Central Pacific region, while the largest comes from South Pacific region. Above ground and below ground stocks could be related to high precipitation and allochthonous C_org_ from river inputs, respectively (Adame & Fry, 2016; Ezcurra et al., 2016). Additionally, dominant riverine-estuarine of the South Pacific region promote a greater contribution of runoff sediments (allochthonous) (Table 3). The lowest below ground C_org_ stocks in the Central Pacific region is related to the small number of studies as well as the geomorphological settings which distress the mangrove distribution and structure (Table 3).

According to the humidity range, the largest total C_org_ storage was recorded for the humid climate at 368 ± 35 Mg C_org_ ha^−1^; the lowest value was located in the arid climate at 196 ± 22 Mg C_org_ ha^−1^. The highest values in the below ground C_org_ storage was from the humid climate at 512.3 ± 53 Mg C_org_ ha^−1^ (Fig. 3B). Regarding the ecological mangrove, the Peten (hammock) type presented the highest C_org_ stock (728 ± 230 Mg C_org_ha^−1^), of which 84% (932 ± 105 Mg C_org_ ha^−1^) belonged to the underground compartment (Fig. 3C). The freshwater inputs (springs) from karst soil fractures favored low water stress and high content of nitrates (Herrera-Silveira, Comin & Capurro-Filograsso, 2013). In contrast, dwarf mangrove exhibited the lowest C_org_ stock value (267 ± 22 Mg C_org_ ha^−1^) associated with locations where low phosphorus content limits the absorption of nutrients, which are scarce due to the absence of external sources such as rivers and the calcareous nature of the rock.

**Figure 4:**
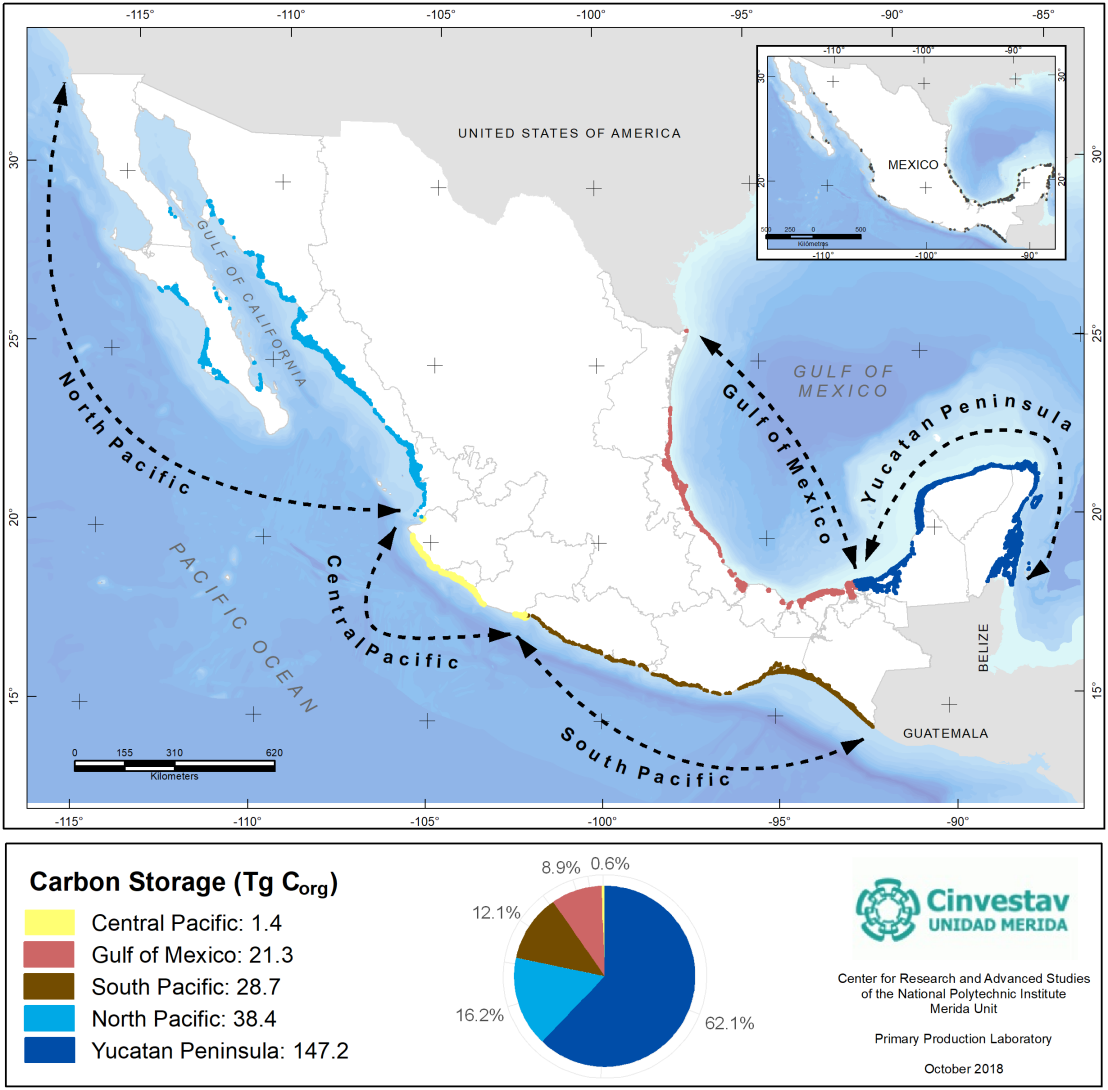
Mangroves C_org_ stocks by geographic regions of Mexico. Data represent the total mangroves pool resulting from the cover extent and regional geographic differences in C_org_ stocks per unit of area. Data units are in Teragrams (1Tg = 1,000,000 Mg). Geographic Coordinate System: WGS 84. Reference cartography of geographic regions and mangrove extent, from INEGI (2011) and CONABIO (2009).

According to the extension and ecosystem C_org_ mean (Tables 1 and 3), the mangroves constitute a reservoir for Mexico of approximately 237.7 Tg C_org_ or 872.3 Tg CO_2_e. By region, the results indicate that the Yucatan Peninsula shows the highest reservoir of C_org_ (148.2 Tg) (Fig. 4) while the Central Pacific region accounts for the smallest C_org_ stocks (1.4 Tg). It is important to mention that the South Pacific has the highest average stock of C_org_ (Table 4), although its mangrove spatial cover is low (Table 4). The importance of mangroves in the Yucatan Peninsula is not related to the forest structure, although they do cover a large area, which constitutes 51% of the total area of mangroves in Mexico. This region is characterized by a low inland topography and high groundwater influence due to the shallow water table (<1 m), thus allowing for the creation of subterranean estuaries that support mangrove over more than 20 km inland (Herrera-Silveira, Comin & Capurro-Filograsso, 2013).

Improving and reforming the policy and legal framework for mangroves from a socioecological point of view is needed in order to restrict and regulate human activities that cause the degradation of these ecosystems. Such restrictions will alleviate the conflict of interest in Mexican coastal zones among conservation and economic activities (mainly aquaculture and tourism).

Although data related to averages and regions are generally reported, it is important to highlight that basic information of C_org_ storage is unknown in mangrove areas. Although averages are used for the estimation of C_org_ at the ecosystem level, the heterogeneity of the data among regions indicates that site-specific evaluations are still required to represent the variability and reduce uncertainty in the estimations (Table 3). This information is essential for including these ecosystems in payment mechanisms for environmental services, as well as voluntary carbon markets (Lau, 2013).

**Table 4:** Summary mangrove and seagrasses C_org_ stocks acording to the cover extent. Estimated mangrove emissions are shown with their associated land use change per geographic region. Mangrove emissions were calculated for the time period 1970-2015 ussing a conservative approach of 25% loss of the total storage. Main factors of change: 1. Aquaculture farms and artificial ponds, 2. Construction areas, 3. Hydraulic infrastructure, 4. Communication routes, 5. Industrial zones and 6. Turistic zones (from Valderrama-Landeros et al., 2017).

	MANGROVES					SEAGRASS	
Region	**Mangrovecover(ha)**	**Carbon Stored(Tg C**_**org**_**)**	**Loss of mangrove area(1970-2015)(ha)**	**Emissions 25%(Tg CO**_**2**_**e)**	Main factors of change*	**Seagrassescover(ha)**	**CarbonStored(Tg C**_**org**_**)**
North Pacific	187,383	38.3	10,512	1.9	1, 4	47,400	1.2
Central Pacific	7,011	1.4	9,464	1.8	2, 4	ND	ND
South Pacific	72,187	28.6	26,563	9.6	3, 5	ND	ND
Gulf of Mexico	87,048	21.2	2,602	0.5	1, 4	341.9	0.02
Yucatan Peninsula	421,926	148.2	31,709	10.1	2, 6	413,317	46.9

### Synthesis of carbon stocks in seagrasses by region

The extent of seagrasses considered in this study was 461,058 ha distributed in three regions of Mexico: Yucatan Peninsula (89.6%), Gulf of Mexico (0.1%) and North Pacific (10.3%). According to the analysis of the data collected from the C_org_ stocks, the average above ground stock per unit area was 0.78 ± 1.19 Mg C_org_ ha^−1^ and129.21 ± 113.4 Mg C_org_ ha^−1^for below ground areas, and the total C_org_ stock for Mexico was 130 Mg C_org_ ha^−1^. This value is slightly lower than that reported by Phang, Chou & Friess (2015) for tropical meadows (138 ± 8.6 Mg C_org_ ha^−1^). The C_org_ stocks from the live biomass of seagrasses have been estimated at 2.52 ± 0.48 Mg C_org_ ha^−1^, of which 75% is composed by roots and rhizomes (Fourqurean et al., 2012a). For sediments, the reported stocks vary from 9.1 to 628.1 Mg C_org_ ha^−1^, with a conservative average of 139.7 Mg C_org_ ha^−1^ (Fourqurean et al., 2012a). The estimations of seagrass C_org_ stocks are highly variable because they depend on the species, the local environment and the seasonality of the survey (Macreadie et al., 2014). In Mexico, the average stock of C_org_ for living above ground (mainly leaves) was 0.78 ± 1.19 Mg C_org_ ha^−1^, although high variability was observed (Table 3).

**Figure 5:**
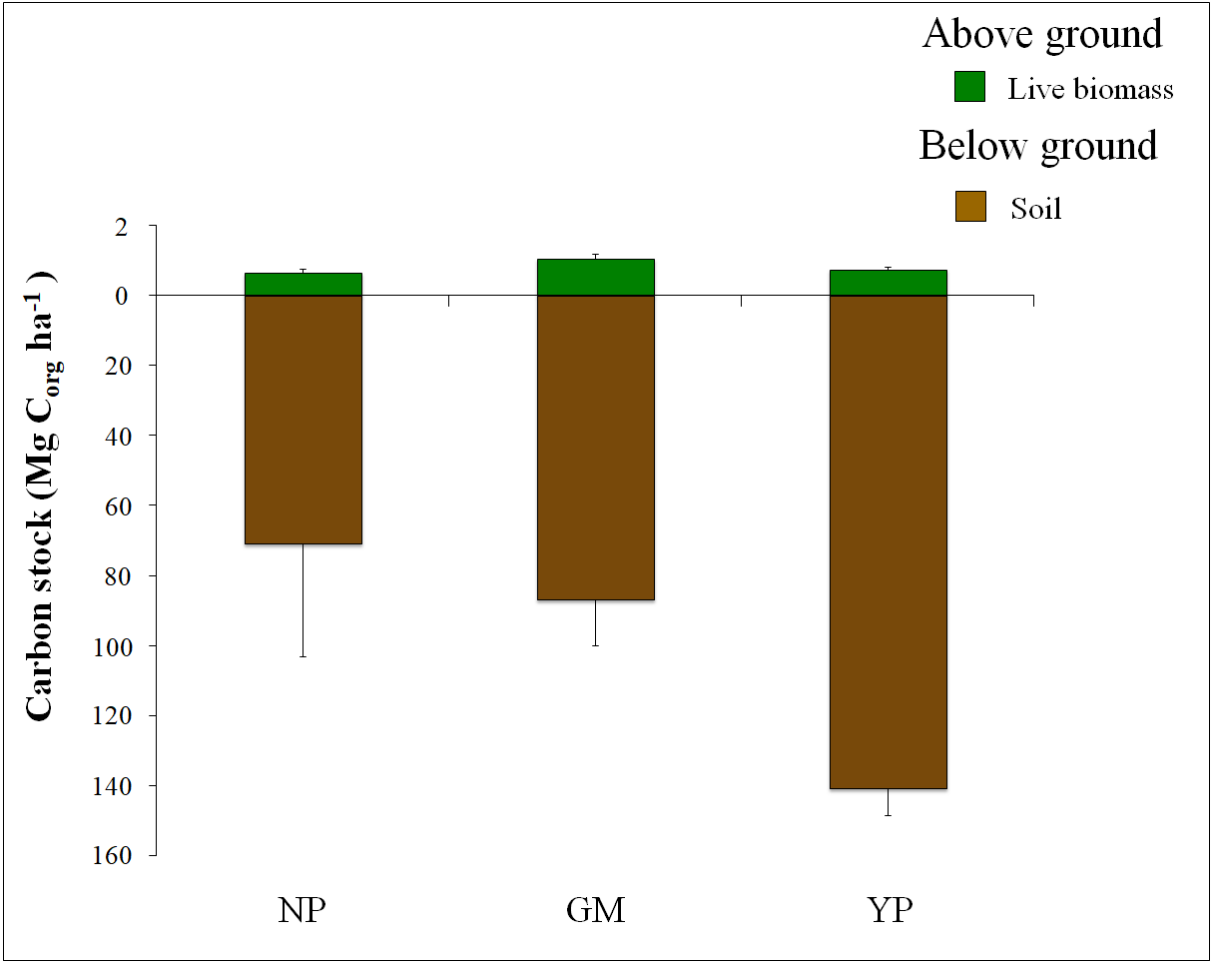
Partitioning of Seagrasses’s C_org_ average stocks in above and below ground for each Mexican geographic region. Mexican seagrasses’s C_org_ stocks per unit of area in Mg ha^−1^. Soil stocks were standarized to 1m depth. The stocks were divided by above and below ground components. Error bars represents SE. Note the different scales used in above and below ground C_org_ stocks.

Regionally, the largest C_org_ stocks per unit area were registered in the Yucatan Peninsula and smaller ones were observed in the North Pacific (Fig. 5) where the greatest value of uncertainty was recorded (Table 3). Our results for Yucatan Peninsula were higher than that reported by Thorhaug et al. (2018) for the Yucatan Peninsula (17.5 Mg C_org_ ha^−1^). The wide continental shelf and coastal geomorphology favored semiclosed or protected water bodies (bays and coastal lagoons) and zones with a low intensity of water currents, as in the north and west coast of the Yucatan Peninsula (Herrera-Silveira, Comin & Capurro-Filograsso, 2013), where the highest total C_org_ stocks (113.7 ± 7 Mg C_org_ ha^−1^) were found. However, the Pacific coast reported the lowest total C_org_ stocks (26.1 ± 13 Mg C_org_ ha^−1^), which was most likely related to the physical characteristics of the region, such as narrow continental shelf and high discharge of rivers that are sources of sediments leading to high turbidity in the coastal waters (De la Lanza Espino, Pérez & Pérez, 2013). The differences in the dominant hydrological conditions of each site could be playing an important role in the carbon reservoir of this region, and they also partially explain the high uncertainty values (24.2% -93.7%) of the seagrasses carbon stocks (Table 3). Regarding to the Gulf of Mexico’s seagrasses, our study reports a total C_org_ stocks (66.1 ± 10 Mg C_org_ ha^−1^) which is higher than reported by Thorhaug et al. (2017) for this area (25.7 ± 17.7 Mg C_org_ ha^−1^).

Efforts to study seagrasses in Mexico extend back to the 1950s; however, it has not been possible to determine the national extent with precision. This is a crucial knowledge gap as changes in extent are required to estimate carbon stocks as well as emissions. The methodological difficulties for characterization of the marine environment in addition to the legal abandonment by the Mexican environmental authorities, are reflected in the variability of the seagrass extensions reported by different Mexican sources (Table 1). The geographic regions with the largest amount of data to estimate the blue carbon storage were the Yucatan Peninsula and the Gulf of Mexico. The Pacific Center was the region with scarce information for blue carbon estimations. The Central Pacific and South Pacific regions did not present representative areas of seagrass distribution (CCA, 2017). In this sense, regionalization allows researchers to distinguish climate, geomorphological, environmental, seagrasses species and human use differences that have not been previously evaluated but certainly are drivers that impact the carbon storage capacity (Table 2). Coastal lagoons and arid climate dominate the North Pacific, while the Central Pacific, Southern Pacific and Gulf of Mexico regions present riverine-estuarine systems with a sub-humid climate as dominant settings; finally, the Yucatan Peninsula region is characterized by karst systems with groundwater discharges (Herrera-Silveira & Morales-Ojeda, 2009; De la Lanza Espino, Pérez & Pérez, 2013). The information generated for blue carbon ecosystems is not homogeneous for C_org_ components (above and below ground) or in the different regions of the country, and research in each region is required to reduce the estimated uncertainty (Tables 2 and 3).

**Figure 6:**
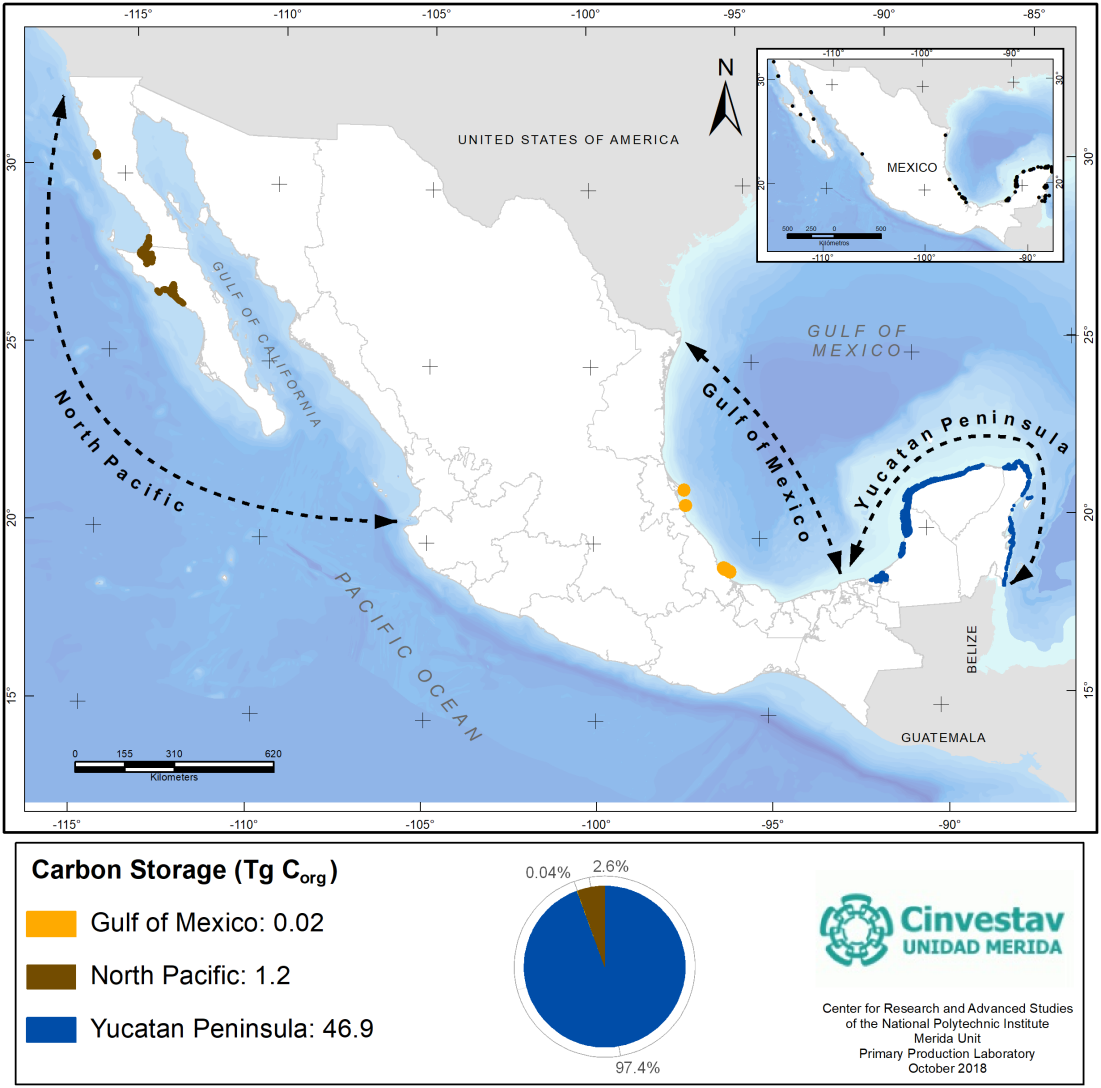
Seagrasses’s C_org_ stocks by geographic regions of Mexico. Data represent the total seagrasses’s pool resulting from the cover extent and regional geographic differences in C_org_ stocks per unit of area. Data units are in Teragrams (1Tg = 1,000,000 Mg). Geographical Coordinates Systems. Datum: DWGS 1984. Reference cartography of geographic regions from : INEGI (2011), and seagrasses extent from Gallegos-Martínez, et al (2017) and Gallegos-Martínez, Hernández-Cárdenas & Pérez Espinosa (2018).

Thus, according to our study, the estimated country stock for seagrasses is 48.1 Tg C_org_ and the Yucatan Peninsula contribution is close to 97% (Fig. 6); therefore, the conservation and application of restrictive policies on activities that impact the seagrasses in this region must be prioritized, particularly due to the conflicts of interest with the tourism sector.

### Mexico’s blue carbon in context

The climate and geomorphology of water bodies along the coasts of each country region have resulted in hydrological differences related to fresh water inputs, hydroperiods, tidal ranges, hydrodynamics, and nutrient supplies and the presence of stressors that could influence the stocks and flux (import/export rates) of C_org_ in mangroves and seagrasses (Woodroffe, 1992; Twilley & Rivera-Monroy, 2005; Fourqurean et al., 2012a; Mazarrasa et al., 2018).

**Figure 7:**
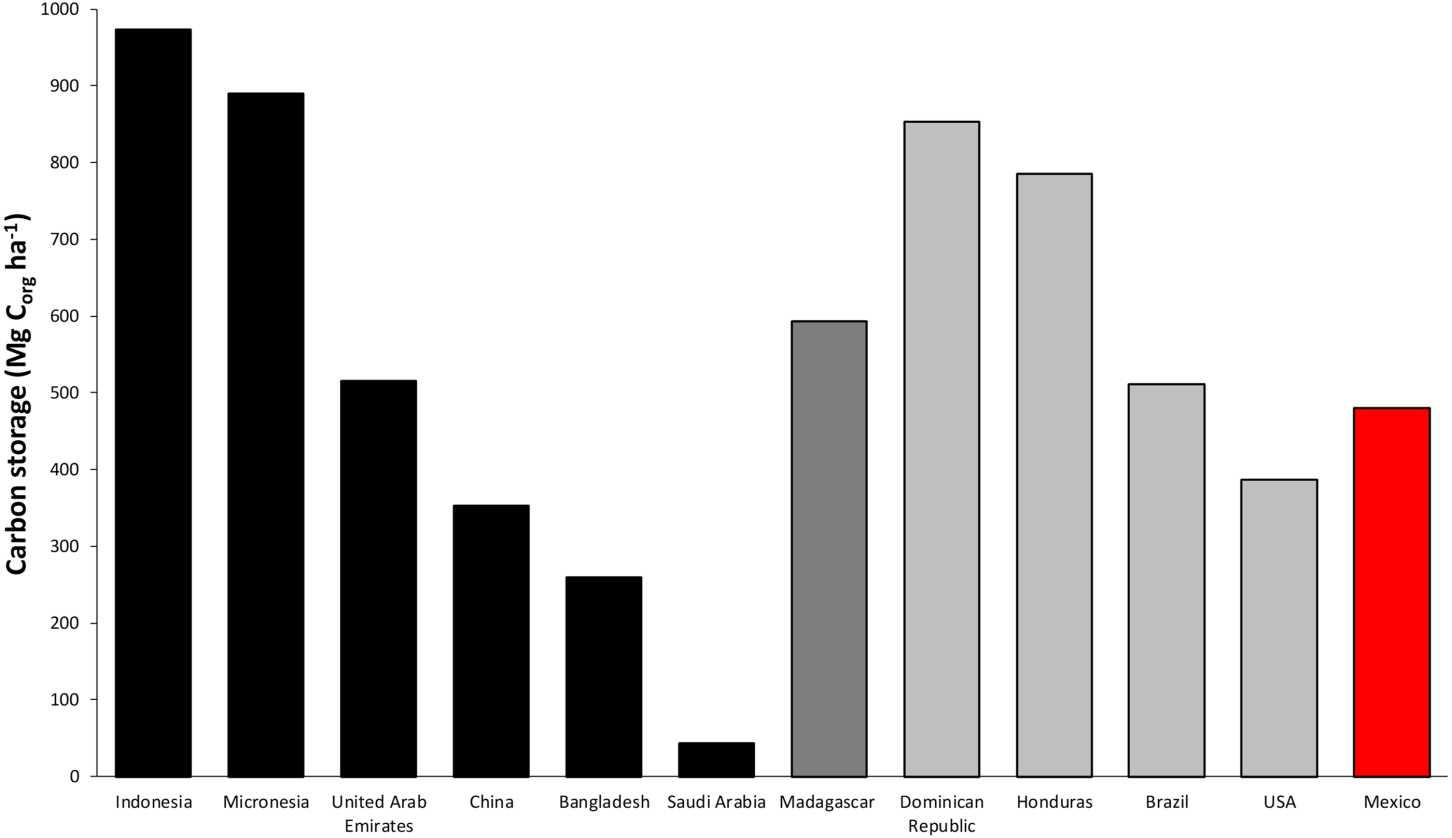
Worldwide average comparison of C_org_ stocks per unit of area in mangroves. Data in Mg per unit of area (ha). Black bars correspond to data from Asia (data reported by Kauffman et al., 2011; Liu et al., 2014; Schile et al., 2017; Rahman et al., 2015 and Almahasheer et al., 2017). Dark gray bars correspond to data from Africa (Jones et al., 2014). Light gray bars correspond to data from America (Kauffman et al., 2014; Bhomia et al., 2016; Kauffman et al., 2018 and Thorhaug et al., 2018). Red bar corresponds to data from Mexico obtained in this study.

For mangroves, the environmental heterogeneity (Table 2) results in equally variable C_org_ stocks ranging from <10 to 2,233 Mg C_org_ ha^−1^. This high variability has been reported around the world (Fig. 7). Low C_org_ stocks characterize the arid region of Saudi Arabia (24.71 Mg C_org_ ha^−1^, (Almahasheer et al., 2017), while high values are often reported for very humid regions, such as Indonesia (1,691 Mg C_org_ ha^−1^, Alongi et al., 2016) and Micronesia (1,385 Mg C_org_ ha^−1^, Kauffman et al., 2011). At the country level, below ground C_org_ stocks values are up to 1,300 Mg C_org_ ha^−1^ (sample depth 2.3 m) in arid climates, such as the Mexican Northern Pacific region according to Ezcurra et al. (2016). Isotope analyses of *δ*^14^C and *δ*^13^C in mangrove soil from the Pacific coast of Mexico suggest that peat formation and vertical accretion tend to develop in topographically constrained mangroves, and most of the soil C_org_ of mangroves seems to derive from *in situ* production in ecosystems without riverine influence (Adame & Fry, 2016; Ezcurra et al., 2016). These findings suggest that in addition to regional characteristics (Table 2), local variables, such as the hydroperiod, allochthonous nutrient inputs, and geomorphological history of the site, play important roles in the sequestration and storage of below ground C_org_ in mangroves. Therefore, considerable work is required to examine the interactions among these variables and the C_org_ measurements of the entire soil profile.

**Figure 8:**
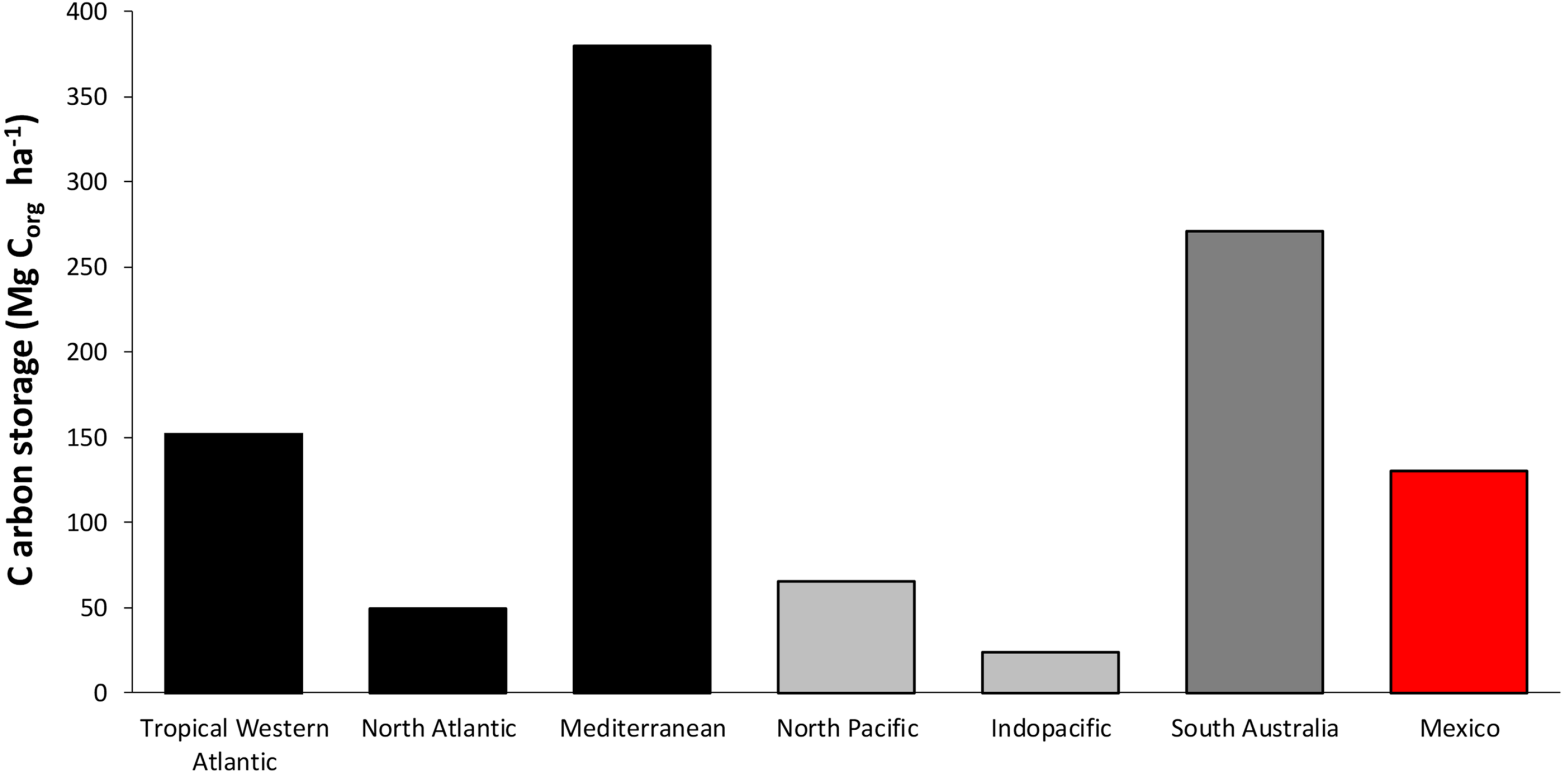
Worldwide average comparison of C_org_ stocks per unit of area in seagrasses. Data in Mg per unit of area (ha). Black bars correspond to average data from sites located in the Atlantic sea region (constructed from Fourqurean et al., 2012a; Fourqurean et al., 2012b data); light gray represent data from Pacific Region sites (Fourqurean et al., 2012a; Fourqurean et al., 2012b); dark gray bar represent data from Australia (Fourqurean et al., 2012a; Fourqurean et al., 2012b) and finally, red bar corresponds to Mexico (total result from the addition of above and below ground total averages).

Regarding seagrasses, Mexico ranks fourth (130 Mg C_org_ ha^−1^) in terms of carbon stock per unit area (ha) (Fig. 8), which is lower than the upper boundary of the global average C_org_ stock described in the literature for the Mediterranean region (375 ± 114 Mg C_org_ ha^−1^) and south Australia (270 Mg C_org_ ha^−1^) (Fourqurean et al., 2012a). An important factor to consider for seagrass carbon stocks at the worldwide level is the longitudinal factor. Short, Short & Novak (2016) reported four temperate regions (including the Mediterranean) and two tropical regions based on assemblages of taxonomic groups and physical separation. These characteristics among others, such as local hydrodynamics, determine the C_org_ sequestration rate and stocks of seagrass meadows (both aerial and sediment). In general, Mediterranean and tropical regions (western Atlantic, south Australia) present optimal storage conditions for large amounts of C_org_ in comparison to temperate regions (north Atlantic, north Pacific). The Mediterranean is a very well-studied region that presents vast deep meadows with a moderate diversity of a temperate/tropical mix of seagrasses (9 species) growing in clear water. There are different factors that could favor the large stocks; for example, the ecological structure or composition, the architecture of the dominant endemic and deep-growing species *Posidonia oceanica*, which forms a root and rhizome “mat” that could be several meters deep and thousands of years old and is an inherent characteristic in addition to the semi closed geomorphology of the Mediterranean that favors the large C_org_ stocks observed in that region.

In Mexico the current extent of seagrasses must be understood as the result of additive factors, such as regional and local environmental particularities and human impacts, on the coastal zone. Environmental heterogeneity represented in the country regions, such as differences in the tidal range, wave energy, transparency, water temperature, salinity and nutrient inputs from inland freshwater (rivers or groundwater), drive seagrasses distribution, composition and structure as well as soil processes that indicate the capacity for capturing carbon by seagrasses. Human activities in the Pacific, Gulf of Mexico and Yucatan Peninsula regions influence the C_org_ stocks, which vary from 26.1 to 113.7 Mg C_org_ ha^−1^ (Table 3). The heterogeneity of Mexican seascapes and hydrogeologic characteristics provided along the 11,000 km of coasts account for coastal lagoons, bays and shallow coastal zones, which are suitable for seagrasses habitat development.

In the Yucatan Peninsula, a wide continental shelf with a low slope and depths of less than 10 m away from the coast to more than 20 km represent the conditions for storing large C_org_ stocks. Conversely, the lower C_org_ stocks are usually located at sites with high hydrodynamic energy, lower transparency and greater seasonal variation in water temperature, such as those observed in the North Pacific region (Arreola-Lizárraga et al., 2018).

These findings support the need for higher-level analyses of the variables related to the capture and storage capacity of C_org_ from blue carbon ecosystems at the locality or specific site scale and even at the global scale. Factors such as climate and geomorphology have been previously identified to be key variables (Sanders et al., 2016; Twilley, Rovai & Riul, 2018). However, in this synthesis, the local scale characteristics related to nutrient inputs and hydrology, especially the time of flooding in mangroves, micro climatic conditions, coast geomorphology, as well as hydrodynamics, dominant species, and type of sediment for seagrasses, suggested that all of these could be important characteristics favoring the variability observed in C_org_ stocks of mangroves and seagrasses. The evaluation of blue carbon ecosystems at the local scale is the baseline, before evaluating the feasibility of including these ecosystems in a national environmental compensation scheme, payment for ecosystem services or carbon markets. In this work, great variability of the C_org_ reservoirs was observed according to the region and climate, and site-specific variables, such as species, temperature, source of water, porewater salinity and hydroperiod, could be associated with their capacity to store and sequester C_org_. The addition of this information will provide a better understanding of the process related to C_org_ sequestration and storage capability, which will be a useful input for direct conservation planning as well as management and restoration plans at country, regional and local levels (Friess et al., 2016).

### Synthesis of the blue carbon fluxes in Mexican coastal ecosystems

For greenhouse gas emissions due to the loss of mangroves, the results showed that the highest emissions occurred in the regions with the greatest loss of mangrove: The Yucatan Peninsula has contributed the most (10.1 Tg CO_2_e), followed by the South Pacific region (9.6 Tg CO_2_e). The Gulf of Mexico has the lowest contribution to CO_2_e emissions due to mangrove loss (0.5 Tg CO_2_e). It is important to highlight that mangrove cover in Central Pacific has shown the greatest loss over the last 45 years; however, its emissions are lower due to the low storage capacity per hectare (Table 3). According to Valderrama-Landeros et al. (2017), the main factors underlying the change in the mangrove ecosystem in Mexico are related to anthropogenic activities, such as aquaculture and coastal urban development (mainly tourism); however, the impact is variable according to the intensity, extent and region in which they develop (Table 4).

Studies related to emissions from mangroves to grassland conversion results in 786 to 2,173 Mg CO_2_e ha^−1^ (Kauffman et al., 2016), which are higher than those estimated in this study due to a more conservative approach (Table 4). However, the mangrove loss due to their conversion to shrimp farms leads to an estimated emission of 2,637 Mg CO_2_e ha^−1^, which represents an increment up to 80% (Kauffman et al., 2014). Therefore, it is important to strengthen the research on the specific impact of the activities carried out in each region, which will allow researchers and resource managers to determine estimated emission factors per activity and identify where the conservation and restoration actions are a priority in order to preserve the ecosystem service of emission avoidance (Teutli-Hernández & Herrera-Silveira, 2016). Mangrove restoration success is largely related to hydrologic improvements, which favor soil accretion rates. The main co-benefits associated with restoration activities are the less vulnerable and more resilient mangroves and human communities to sea level rise and the enhancement of biodiversity, including the reincorporation of ecologically and commercially important species of mollusk, fish, and birds (Arceo-Carranza et al., 2016).

For seagrasses, disturbance represents a significant loss of the total carbon stock, which contributes to CO_2_e emissions due to the argumentation of oxidative processes of the organic matter (Moodley et al., 2005) by unburied sediment (Marbà et al., 2015). Few studies have been conducted to assess the contribution of CO_2_e emissions to the atmosphere attributed to the loss of the seagrass cover, despite the high worldwide deterioration of seagrasses (Waycott et al., 2009) over recent decades. Nevertheless, a loss of 2.63% and up to 3.37% (Lovelock, Fourqurean & Morris, 2017) of the total stock has been estimated due to the loss of seagrass cover resulting from water quality deterioration and beach erosion, respectively.

Based on the average values of CO_2_e emissions reported in the literature, and due to the lack of direct measures of this process in Mexican seagrasses ecosystems, our first approach to examining potential emissions for seagrasses ranges from 5.81 to 7.44 Tg CO_2_e considering the loss of 463,240.6 ha of seagrasses according to the extensions reported over 12 years (2006-2018); however, this value should be interpreted with caution due to the lack of systematic monitoring of the seagrass cover. The incorporation of remote sensing in seagrass surveys and mapping methodologies (Mendoza-Martínez, 2017; Cerdeira-Estrada et al., 2018) as well as the development of new and less expensive remote sensors or equipment such as SENTINEL 2a and unmanned aircraft system (drone) make it possible to improve the distribution maps of marine landscapes in the near future.

An important issue is the vertical and lateral carbon flux such as the capture and exchange of methane (CH_4_), particulate organic carbon (POC) and dissolved organic carbon (DOC), which are currently carried out in mangroves. Nevertheless, carbon flux is estimated via the measurement of ecosystem productivity, and despite mangroves being key suppliers of organic carbon to the adjacent coastal systems and being the basis of the theory of “outwelling” proposed by Odum & Heald (1972), the number of quantitative estimations remains scarce.

In a review of 16 studies on mangrove litterfall in Mexico, the average estimated was 4.3 ± 0.6 Mg C_org_ ha^−1^ y^−1^ (Herrera-Silveira et al., 2016), which is close to the estimated interval from global litterfall mangrove measurements ranging from 4.3–4.8 Mg C_org_ ha^−1^ y^−1^ (Hutchison et al., 2014; Zhang et al., 2014). Regionally, the highest average occurred in the South Pacific (6.7 Mg C_org_ ha^−1^ y^−1^), while the lowest was recorded in the Central Pacific (1.5 Mg C_org_ ha^−1^ y^−1^). Air temperature and precipitation are the main variables related to these regional differences as litterfall production is coupled to rain (Santini et al., 2015).

In Mexico, predicting changes in litterfall associated with environmental variations or land use is difficult. Flux studies associated with litterfall that have reported environmental settings that include the hydroperiod, porewater or soil characteristics and macro and microbiota information are scarce. The environmental context as well as certain biota groups, such as bacteria, fungus, crabs, or worms, could determine the litterfall C_org_ flux. Hydroperiod is considered the main source of variation for litter in fringe mangrove forests (Odum, McIvor & Smith, 1982; Twilley, Lugo & Patterson-Zucca, 1986), but detritivore biota such as crabs and bacteria play a similar role in the inland wetlands. It was estimated that litter removal by crabs could be 33 to 77% of the total annual litterfall from the forest floor, promoting low standing stocks of litter and fast rates of leaf removal (Robertson & Daniel, 1989). Thus, in addition to the importance for C_org_ flux, understanding the environmental and biological interactions regulating litterfall decomposition will also help us to comprehend the role of soil organic matter in accretion and accumulation processes in the face of sea level rise (Lovelock et al., 2015).

Regarding mangrove swamps as a source of CH_4_ emissions, only one study has been conducted in tropical coastal lagoons in the Yucatan Peninsula region (Chuang et al., 2017). The findings revealed diffuse fluxes of CH_4_ from the surface water of the lagoon to the atmosphere that varied between 1.3 × 10^−4^ and 0.876 Mg CH_4_ha^−1^ y^−1^, suggesting that coastal lagoons surrounded by mangroves could be important natural sources of CH_4_. Another aspect to consider is that CH_4_ emissions may be greater in mangroves under disturbance conditions (Pendleton et al., 2012).

In relation to the exchange of DOC and POC between the mangrove and the adjacent water body (river, coastal lagoon, sea), the generated data remain scarce except for the study by Camacho-Rico (2018), who reported exchange values between the fringe mangrove and a coastal lagoon of the Yucatan Peninsula region of 6.8 g DOC m^2^ y^−1^ and 76.7 g DOC m^2^ y^−1^. This range of DOC exchange values is based on values reported for karst sites (≈ 0.46 to -108 g DOC m^2^ y^−1^) (Adame & Lovelock, 2011). Regarding the POC, the exportation values (59.1 ± 88 g POC m^−2^ y^−1^) were lower than the average estimated in a mangrove located in river geomorphological areas, such as Papua New Guinea (285 g POC m^−2^ y^−1^) (Robertson & Alongi, 1995). The variability observed in the DOC and POC exchange data is related to the hydrological, climatic, and biotic differences that occur within the water column at each site as well as the different methodologies used.

Most of the studies in Mexico lack environmental information (interstitial salinity, soil nutrients, hydroperiod), and the role of the freshwater inputs via groundwater is poorly understood. Environmental information could explain geochemical processes in soil and plant exposition to stressors resulting in changes in the flux and stocks of organic carbon (Day et al., 1996; Shunula & Whittick, 1999b; Coronado-Molina et al., 2012).

### Mexican policy for blue carbon ecosystems

In the face of climate change, Mexico has joined international efforts to protect and restore blue carbon ecosystems (UNFCCCV, 2015). In this sense, knowledge of the benefits provided by the sequestration and storage of carbon in mangroves and seagrasses as well as the amount of environmental services they provide represents baseline information that is required for the planning, management and prioritization of activities related to the conservation and restoration of these coastal ecosystems.

Mexico has ratified the United Nations Framework Convention on Climate Change (UNFCCC) the agreement, which includes coastal blue carbon ecosystems as climate mitigation and adaptation solutions in its National Determined Contributions (NDCs) (Martin et al., 2016). Even in Mexico’s NDCs, the term “blue carbon” is not mentioned itself; however, the federal government considers coastal wetlands as part of its general mitigation aims, recognizes the benefits of coastal wetlands in the mitigation and adaptation of GHGs, and includes coastal wetlands as adaptation solutions. Mexican NDCs actions include the conservation, management, protection and restoration of wetlands, such as mangroves, seagrass and other coastal and marine ecosystems, which are also considered in conservation and recovery schemes. In Mexico, the implications and capabilities of reporting mangrove and seagrasses carbon storage by the official institution (Comision Nacional Forestal, CONAFOR) through the national greenhouse gas inventory report is currently being evaluated by the federal government, which would position Mexico at the level of Australia in the blue carbon mitigation NDCs context.

At the national level, mangroves and seagrasses (DOF, 2010) are immersed in different conservation schemes, and their management is controlled mainly by the federal government via different agencies with interventions directly or indirectly in these ecosystems, such as the Ministry of Environment and Natural Resources (SEMARNAT), National Water Commission (CONAGUA), and National Forestry Commission (CONAFOR), among others. Regardless, mangrove and seagrasses losses caused by anthropogenic activities are increasing due to gaps and legal contradictions that allow land use changes mainly for road construction and the development of economic activities of high economic impact, such as aquaculture and tourism.

Mexican legislation has recently established national policy instruments on climate change (DOF, 2010), which include prioritizing the sectors with the greatest emission reduction potential. The 5th Communication of Mexico to the IPCC was a precedent for blue carbon ecosystems as an important component of GHG emission reduction policies. Such work has permeated to the research priorities in Mexico, and scientific work related to blue carbon has grown from 2013, including the important elements included in this work. However, the first actions to harmonize the instruments of public policy based on science and compliance at the three levels of government are just taking place. The sites around natural protected areas seem to be natural pilot sites for the implementation of conservation projects and restoration of blue carbon ecosystems as determined by the consensus of authorities, users, academia and NGOs. Studies in mangrove protected areas have revealed high C_org_ stocks (from 663 to 1, 358 Mg C_org_ ha^−1^) (Adame et al., 2013; Adame et al., 2013b; Adame et al., 2015; Kauffman et al., 2016). However, it is important to consider that not all protected natural areas are effective or sufficient given the magnitude of biological diversity, competence and need to maintain other land uses (Ceccom & Martínez-Garza, 2016).

A national regulatory framework offers opportunities for the creation of public mitigation policies aimed to quantify and check the GHG emissions, as well as direct and promote actions to increase carbon budget, stop and reverse coastal ecosystems deforestation and degradation. The incorporation of the concept “blue carbon economy” in the public policy related to climate change through mechanisms based on the carbon market (credits or emissions rights) generated by restoration or conservation of wetlands is an attractive option for the financing of blue carbon projects in Mexico (CCA, 2017).

The variability in C_org_ stocks in mangroves and seagrasses of Mexico observed along the coasts and the uncertainty associated could limit their incorporation to ongoing prospections related to the setting of national baselines under REDD^+^ nesting and feasibility analyses of voluntary local-regional markets (Bejarano, López & Rosette, 2018). In this sense, the risk of following REDD^+^ strategy due to the “additionality” problem must be evaluated at the national level due to Certified Emission Reductions from projects which create ’additional’ emissions reductions to those that would otherwise have been achieved. Blue carbon offsets come with high uncertainties and risks of reversal, and because their additionality (i.e., whether emissions reductions or removals would have happened without the blue carbon project) can be difficult to prove in zones with a high occurrence of natural impacts (i.e., fires, hurricanes, etc.), such as Mexico and the Caribbean Basin. Possible solutions to additionality have been largely discussed, (i.e., use of carbon fluxes or sequestration instead of carbon storage), and the existing information of C_org_ fluxes for Mexican blue carbon ecosystems could not support public policies in the short term for the development of local, regional and national strategies to achieve the international commitments of Mexico.

In Mexico, the incorporation of alternative schemes to incentivize the protection of blue carbon ecosystems, such as the “payment for ecosystem services”, or “national blue carbon markets”, including concessions to communities through custody schemes, could be better implemented and justified by the potential co-benefits from coastal ecosystem conservation and restoration. Then, successful and operative schemes should be designed with the full set of ecosystem services in mind and not just carbon sequestration.

## Conclusions

Mexico has great potential to contribute to mitigating the effects of climate change through the conservation and restoration of blue carbon ecosystems, especially mangroves and seagrasses. This study contributes to the quantification of C_org_ stocks and CO_2_e emissions estimation due to the loss of mangrove cover, and it provides the first data for C_org_ stocks and emissions of seagrasses at the country level.

According to this synthesis, the total carbon storage of Mexican mangroves and seagrasses is 498.8 Mg C_org_ ha^−1^ and 130 Mg C_org_ ha^−1^, respectively. Considering the official extension of both ecosystems, mangroves have a stock of 237.7 Tg C_org_ and seagrasses have a stock of 48.1 Tg C_org_. Together, the blue carbon stock of Mexico compensates for the emissions of ≈300 million hydrocarbon users in a year.

Mexican mangroves exhibit great variability in organic carbon reservoirs according to the region, climate, and vegetation structure. According to the criteria, the highest averages for mangroves by region were exhibited in the South Pacific (397.1 ± 45 Mg C_org_ ha^−1^), in humid climates (368.3 ± 35 Mg C_org_ ha^−1^) and peten vegetation (728 ± 230 Mg C_org_ ha^−1^). The Yucatan Peninsula has the largest regional extension of mangroves (55%), and all structural vegetation typologies are presented; however, this region is the most impacted by land use changes.

Conservation and restoration should be prioritized in this region to avoid emissions, reduce vulnerability to sea level rises, and create adaptation opportunities based on ecosystem management. In the case of seagrasses, we report only for the North Pacific, Gulf of Mexico and Yucatan Peninsula, with the latter presenting the highest storage records (113.7 ± 7 Mg C_org_ ha^−1^).

Coverage loss of blue carbon ecosystems in Mexico has accounted for approximately 24 Tg CO_2_e emissions by mangroves over the last 20 years. However, no coverage change data are available for seagrasses, although preliminary results using literature scenarios and local data indicate emissions of approximately 6 Tg CO_2_e over 12 years. According to these results and if the current loss rates are maintained, the commitments of the Paris agreement signed by Mexico government will be difficult to fulfill.

The results of this synthesis suggest that the policies for conservation and restoration of blue carbon ecosystems in Mexico must be improved and enforced in addition to the current legal framework of protection, which do not properly fulfill their purpose. Official data report tourism, agriculture and land use change as the activities with deleterious impacts on Mexican blue carbon ecosystems. We recommend the regulation of those activities according to ecohydrological approaches in order to improve conservation and connectivity between ecosystems. Such action plans should use blue carbon conservation and restoration as an umbrella to maintain and/or recover environmental services, at the time synergies among government, academic, social and private sectors are reinforced and harmonized by win-win initiatives.

##  Supplemental Information

10.7717/peerj.8790/supp-1 Data S1 Information collected about the carbon stored in the mangroves of Mexico

10.7717/peerj.8790/supp-2 Data S2 Information collected about the carbon stored in the seagrasses of Mexico

10.7717/peerj.8790/supp-3 Table S1 Carbon storage in mangroves by ecological type and weather Columns shows aboveground carbon average, belowground carbon average and total carbon average, with respective standard errors, minims and maxims for mangroves.

10.7717/peerj.8790/supp-4 Supplemental Information 4 Rationale and contribution

10.7717/peerj.8790/supp-5 Supplemental Information 5 PRISMA checklist

## Additional Information and Declarations

### Data Availability

The following information was supplied regarding data availability: The raw measurements are available in the Supplemental Files.

## References

[ref-1] Adame MF, Brown CJ, Bejarano M, Herrera-Silveira JA, Ezcurra P, Kauffman JB, Birdsey R (2018). The undervalued contribution of mangrove protection in Mexico to carbon emission targets. Conservation Letters.

[ref-2] Adame MF, Fry B (2016). Source and stability of soil carbon in mangrove and freshwater wetlands of the Mexican Pacific coast. Wetlands Ecology and Management.

[ref-3] Adame MF, Kauffman JB, Medina I, Gamboa JN, Torres O, Caamal JP, Reza M, Herrera-Silveira JA (2013a). Carbon Stocks of Tropical Coastal Wetlands within the Karstic Landscape of the Mexican Caribbean. PLOS ONE.

[ref-4] Adame MF, Lovelock CE (2011). Carbon and nutrient exchange of mangrove forests with the coastal ocean. Hydrobiologia.

[ref-5] Adame MF, Santini NS, Tovilla C, Vázquez-Lule A, Castro L, Guevara M (2015). Carbon stocks and soil sequestration rates of riverine mangroves and freshwater wetlands. Biogeosciences.

[ref-6] Adame MF, Teutli C, Santini NS, Caamal JP, Zaldívar-Jiménez A, Hernández R, Herrera-Silveira JA (2014). Root biomass and production of mangroves surrounding a karstic oligotrophic coastal lagoon. Wetlands.

[ref-7] Adame MF, Zaldívar-Jimenez A, Teutli C, Caamal JP, Andueza MT, López-Adame H, Cano R, Hernández-Arana HA, Torres-Lara R, Herrera-Silveira JA (2013b). Drivers of mangrove litterfall within a karstic region affected by frequent hurricanes. Biotropica.

[ref-8] Almahasheer H, Serrano O, Duarte CM, Arias-Ortiz A, Masque P, Irigoien X (2017). Low carbon sink capacity of Red Sea mangroves. Scientific Reports.

[ref-9] Alongi DM (2008). Mangrove forests: resilience, protection from tsunamis, and responses to global climate change. Estuarine, Coastal and Shelf Science.

[ref-10] Alongi DM (2012). Carbon sequestration in mangrove forests. Carbon Management.

[ref-11] Alongi DM, Murdiyarso D, Fourqurean JW, Kauffman JB, Hutahaean A, Crooks S, Lovelock CE, Howard J, Herr D, Fortes M, Pidgeon E, Wagey T (2016). Indonesia’s blue carbon: a globally significant and vulnerable sink for seagrass and mangrove carbon. Wetlands Ecology and Management.

[ref-12] Arceo-Carranza D, Gamboa E, Teutli-Hernández C, Badillo-Alemán M, Herrera-Silveira JA (2016). Los peces como indicador de restauración de áreas de manglar en la costa norte de Yucatán. Revista Mexicana de Biodiversidad.

[ref-13] Arreola-Lizárraga JA, Padilla-Arredondo G, Ruiz-Ruiz TM, Cruz-García LM, Méndez-Rodríguez LC, Hernández-Almaraz P, Vargas-González HH, Ortega-Rubio A (2018). Estuaries and coastal lagoons of mexico: challenges for science, management, and conservation. Mexican natural resources management and biodiversity conservation: recent case studies.

[ref-14] Bejarano M, López E, Rosette M (2018). Análisis de la Situación Política y Económica del Carbono Azul en México. PNUD CSP-2016-057. Programa Mexicano del Carbono, Pronatura Sur, FMCN, CEMDA, CINVESTAV-IPN.

[ref-15] Bhomia RK, Mackenzie RA, Murdiyarso D, Sasmito SD, Purbopuspito J (2016). Impacts of land use on indian mangrove forest carbon stocks: implications for conservation and management. Ecological Applications.

[ref-16] Bouillon S, Connolly RM, Nagelkerken I (2009). Carbon exchange among tropical coastal ecosystems. Ecological connectivity among tropical coastal ecosystems.

[ref-17] Caamal-Sosa JP, Zaldívar A, Adame-Vivanco F, Teutli-Hernández C, Andueza MT, Pérez R, Herrera-Silveira JA (2011). Almacenes de carbono en diferentes tipos ecológicos de manglares en un escenario cárstico. Estado Actual del Conocimiento del Ciclo del Carbono y sus Interacciones en México: Síntesis a.

[ref-18] Camacho-Rico A (2018). Dinamica de intercambio de carbono y nutrientes entre el manglar y la Laguna costera de Celestun, Yucatan. Thesis.

[ref-19] Canty A, Ripley BD (2019).

[ref-20] Commission for Environmental Cooperation (CCA) (2016). Comision para la Cooperacion Ambiental. Carbono azul en América del Norte: evaluación de la distribución de los lechos de pasto marino, marismas y manglares, y su papel como sumideros de carbono.

[ref-21] Commission for Environmental Cooperation (CCA) (2017). Comision para la Cooperacion Ambiental. Análisis de las oportunidades para la integración del concepto de carbono azul en la política pública mexicana.

[ref-22] Ceccom E, Martínez-Garza C (2016). Experiencias mexicanas en la restauracion de los ecosistemas.

[ref-23] Cerdeira-Estrada S, Rosique-De La Cruz LO, Blanchon P, Uribe-Martínez A, Martell-Dubois R, Martínez-Clorio MI, Cruz-López MI, Ressl R (2018). Relieve de los Ecosistemas Marinos del Caribe Mexicano: Cabo Catoche—Xcalak, Escala 1: 8000.

[ref-24] Chuang PC, Young MB, Dale AW, Miller LG, Herrera-Silveira JA, Paytan A (2017). Methane fluxes from tropical coastal lagoons surrounded by mangroves, Yucatán, Mexico. Journal of Geophysical Research: Biogeosciences.

[ref-25] Cinco-Castro S, Camacho-Rico A, Morales-Ojeda S, Caamal-Sosa JP, Herrera-Silveira JA (2017). Almacenes de carbono en humedales costeros del Pacifico Norte y Península de Yucatán. Estado Actual del Conocimiento del Ciclo del Carbono y sus Interacciones en México.

[ref-26] CONABIO (2009). Manglares de México: Extensión y distribución. 2nd edition.

[ref-27] CONABIO (2016). Distribución de los manglares en México en 2015, escala: 1:50000. 1st edition.

[ref-28] CONABIO (2018). Comisión Nacional para el Uso y Conocimiento de la Biodiversidad y Universidad Autónoma Metropolitana. 1st edition.

[ref-29] Coronado-Molina C, Alvarez-Guillen H, Day JW, Reyes E, Perez BC, Vera-Herrera F, Twilley R (2012). Litterfall dynamics in carbonate and deltaic mangrove ecosystems in the Gulf of Mexico. Wetlands Ecology and Management.

[ref-30] Day JW, Coronado-Molina C, Vera-Herrera FR, Twilley R, Rivera-Monroy VH, Alvarez-Guillen H, Day R, Conner W (1996). A 7 year record of above-ground net primary production in a southeastern Mexican mangrove forest. Aquatic Botany.

[ref-31] DOF (2010). Diario Oficial de la Federación. Norma Oficial Mexicana NOM-059-SEMARNAT-2010, que establece las especificaciones para la protección ambiental y de Especies nativas de México de flora y fauna silvestres, Categorías de riesgo y especificaciones para su inclusión, exclusión o cambio de Lista de especies en riesgo.

[ref-32] Donato DC, Kauffman JB, Murdiyarso D, Kurnianto S, Stidham M, Kanninen M (2011). Mangroves among the most carbon-rich forests in the tropics. Nature Geoscience.

[ref-33] Duarte CM, Losada IJ, Hendriks IE, Mazarrasa I, Marbà N (2013). The role of coastal plant communities for climate change mitigation and adaptation. Nature Climate Change.

[ref-34] Ezcurra P, Ezcurra E, Garcillán PP, Costa MT, Aburto-Oropeza O (2016). Coastal landforms and accumulation of mangrove peat increase carbon sequestration and storage. Proceedings of the National Academy of Sciences of the United States of America.

[ref-35] Food and Agriculture Organization of the United Nations (FAO) (2007). The world’s mangroves 1980-2005. FAO Forestry Paper.

[ref-36] Flores MG, Jiménez J, Madrigal X, Moncayo F, Takaki F (1971). Memorias del mapa de tipos de vegetación de la República Mexicana. Secretaria de Recursos Hidráulicos Subsecretaria de planeación Direccion General de Estudios Dirección de Agrología (manual and map scale 1:2,000,000).

[ref-37] Fourqurean JW, Duarte CM, Kennedy H, Marbà N, Holmer M, Mateo MA, Apostolaki ET, Kendrick GA, Krause-Jensen D, McGlathery KJ (2012a). Seagrass ecosystems as a globally significant carbon stock. Nature Geoscience.

[ref-38] Fourqurean JW, Kendrick GA, Collins LS, Chambers RM, Vanderklift MA (2012b). Carbon, nitrogen and phosphorus storage in subtropical seagrass meadows: examples from Florida Bay and Shark Bay. Marine and Freshwater Research.

[ref-39] Friess DA, Thompson BS, Brown B, Amir AA, Cameron C, Koldewey HJ, Sasmito SD, Sidik F (2016). Policy challenges and approaches for the conservation of mangrove forests in Southeast Asia. Conservation Biology.

[ref-40] Gallegos-Martínez M, Hernández-Cardenas G, Espinosa IPérez, Andreas-Ressl R (2017). Comunidad de Pastos marinos del Caribe Mexicano, 2017. Comisión Nacional para el Uso y Conocimiento de la Biodiversidad y Universidad Autónoma Metropolitana, CONABIO-UAM.

[ref-41] Gallegos-Martínez M, Hernández-Cárdenas G, Pérez Espinosa I (2018). Pastos marinos del Estado de Veracruz, México, escala: 1:1. edición: 1. Universidad Autónoma Metropolitana Unidad Iztapalapa e Instituto Nacional de Ecología y Cambio Climático. Proyecto financiado por ’Indicadores del estado de las comunidades de Pastos Marinos en la zona costera del Golfo de México susceptible de ser impactada por los hidrocarburos derramados por la Plataforma Horizon operada por BP’.

[ref-42] García E (1988). Modificación al Sistema de Clasificación Climática de KÖPEN para adaptarlo a las condiciones de la República Mexicana. Cuarta edición.

[ref-43] García E (1990). Rangos de humedad. Extraido de Climas. IV.4.10. Atlas Nacional de México. Vol II. Escala 1: 4000000.

[ref-44] Gautier D, Amador J, Newmark F (2001). The use of mangrove wetland as a biofilter to treat shrimp pond effluents: preliminary results of an experiment on the Caribbean coast of Colombia. Aquaculture Research.

[ref-45] Giri C, Ochieng E, Tieszen LL, Zhu Z, Singh A, Loveland T, Masek J, Duke N (2011). Status and distribution of mangrove forests of the world using earth observation satellite data. Global Ecology and Biogeography.

[ref-46] Green EP, Short FT (2003). World atlas of seagrasses.

[ref-47] Herrera-Silveira JA, Camacho-Rico A, Caamal-Sosa J, Cinco-Castro S, Morales-Ojeda S, Ramírez-Ramírez J, Zenteno-Díaz K, Pech-Poot E, Pech-Cárdenas M, Carrillo-Baeza L, Erosa-Angulo J, Pérez-Martínez O, Teutli-Hernández C (2018a). Database of carbon stocks in the mangroves of mexico. Elementos para Políticas Públicas.

[ref-48] Herrera-Silveira JA, Camacho Rico A, Pech E, Pech M, Ramírez Ramírez J, Teutli Hernández C (2016). Dinámica del carbono (almacenes y flujos) en manglares de México. Terra Latinoamericana.

[ref-49] Herrera-Silveira JA, Comin FA, Capurro-Filograsso L, Day JW, Yáñez Arancibia A (2013). Landscape, Land-Use, and Management in The Coastal Zone of Yucatan Peninsula. Gulf of Mexico: origin, waters, and biota.

[ref-50] Herrera-Silveira JA, Mendoza-Martínez J, Morales-Ojeda S, Camacho-Rico A, Medina-Gómez I, Ramírez-Ramírez I, López-Herrera M, Pech-Poot E, Pérez-Martínez O, Pech-Cárdenas M, Cota-Lucero T, Teutli-Hernández C (2018b). Database of carbon stocks in seagrasses of mexico. Elementos para Políticas Públicas.

[ref-51] Herrera-Silveira JA, Morales-Ojeda SM (2009). Evaluation of the health status of a coastal ecosystem in southeast Mexico: Assessment of water quality, phytoplankton and submerged aquatic vegetation. Marine Pollution Bulletin.

[ref-52] Hiraishi T, Krug T, Tanabe K, Srivastava N, Baasansuren J, Fukuda M, Troxler TG (2014). 2013 supplement to the 2006 IPCC guidelines for national greenhouse gas inventories: Wetlands.

[ref-53] Howard J, Hoyt S, Isensee K, Telszewski M, Pidgeon E (2014). Coastal Blue Carbon: Methods for assessing carbon stocks and emissions factors in mangroves, tidal salt marshes, and seagrasses.

[ref-54] Hutchison J, Manica A, Swetnam R, Balmford A, Spalding M (2014). Predicting global patterns in mangrove forest biomass. Conservation Letters.

[ref-55] IEA (2014). The power of transformation: wind, sun and the economics of flexible power systems.

[ref-56] INECC (2018). Instituto Nacional de Ecología y Cambio Climático. Sexta Comunicación Nacional y Segundo Informe Bienal de Actualización ante la Convención Marco de las Naciones Unidas sobre el Cambio Climático México. Secretaría de Medio Ambiente y Recursos Naturales.

[ref-57] INEGI (2011). Instituto Nacional de Estadística, Geografía e Informática. Conjunto de Datos Vectoriales y Toponimias de límite Nacional y entidades federativas de México escala. 1. 1000000.

[ref-58] INEGI (2014). Instituto Nacional de Estadística y Geografía.

[ref-59] Intergubernamental Pannel of Climate Change (IPCC) (2014). Climate Change 2014—Synthesis Report.

[ref-60] Jones TG, Ratsimba HR, Ravaoarinorotsihoarana L, Cripps G, Bey A (2014). Ecological variability and carbon stock estimates of mangrove ecosystems in northwestern Madagascar. Forests.

[ref-61] Kathiresan K, Rajendran N (2005). Coastal mangrove forests mitigated tsunami. Estuarine, Coastal and Shelf Science.

[ref-62] Kauffman JB, Bernardino AF, Ferreira TO, Giovannoni LR, De O, Gomes LE, Romero DJ, Jimenez LCZ, Ruiz F (2018). Carbon stocks of mangroves and salt marshes of the Amazon region, Brazil. Biology Letters.

[ref-63] Kauffman JB, Bhomia RK (2017). Ecosystem carbon stocks of mangroves across broad environmental gradients in West-Central Africa: global and regional comparisons. PLOS ONE.

[ref-64] Kauffman JB, Donato D (2012). Protocols for the measurement, monitoring and reporting of structure, biomass and carbon stocks in mangrove forests. Center for International Forestry. Working Paper 86. CIFOR. Bogor, Indonesia.

[ref-65] Kauffman JB, Heider C, Cole TG, Dwire KA, Donato DC (2011). Ecosystem carbon stocks of micronesian mangrove forests. Wetlands.

[ref-66] Kauffman JB, Heider C, Norfolk J, Payton F (2014). Carbon stocks of intact mangroves and carbon emissions arising from their conversion in the Dominican Republic. Ecological Applications.

[ref-67] Kauffman JB, Hernandez Trejo H, Del Carmen Jesus Garcia M, Heider C, Contreras WM (2016). Carbon stocks of mangroves and losses arising from their conversion to cattle pastures in the Pantanos de Centla, Mexico. Wetlands Ecology and Management.

[ref-68] Koch EW, Barbier EB, Silliman BR, Reed DJ, Perillo GME, Hacker SD, Granek EF, Primavera JH, Muthiga N, Polasky S (2009). Non-linearity in ecosystem services: temporal and spatial variability in coastal protection. Frontiers in Ecology and the Environment.

[ref-69] Koricheva J, Gurevitch J, Mengersen K (2013). Handbook of meta-analysis in ecology and evolution.

[ref-70] De la Lanza Espino G, Pérez MAO, Pérez JLC (2013). Diferenciación hidrogeomorfológica de los ambientes costeros del Pacífico, del Golfo de México y del Mar Caribe. Investigaciones Geográficas, Boletín del Instituto de Geografía.

[ref-71] Lau WWY (2013). Beyond carbon: conceptualizing payments for ecosystem services in blue forests on carbon and other marine and coastal ecosystem services. Ocean & Coastal Management.

[ref-72] Liu J, Scheuer E, Dibb J, Ziemba LD, Thornhill KL, Anderson BE, Wisthaler A, Mikoviny T, Devi JJ, Bergin M, Weber RJ (2014). Brown carbon in the continental troposphere. Geophysical Research Letters.

[ref-73] Lovelock CE, Adame MF, Bennion V, Hayes M, Reef R, Santini N, Cahoon DR (2015). Sea level and turbidity controls on mangrove soil surface elevation change. Estuarine, Coastal and Shelf Science.

[ref-74] Lovelock CE, Fourqurean JW, Morris JT (2017). Modeled CO2 emissions from coastal wetland transitions to other land uses: tidal marshes, mangrove forests, and seagrass beds. Frontiers in Marine Science.

[ref-75] Lugo AE, Snedaker SC (1974). The ecology of mangroves. Annual Review of Ecology and Systematics.

[ref-76] Macreadie PI, Baird ME, Trevathan-Tackett SM, Larkum AWD, Ralph PJ (2014). Quantifying and modelling the carbon sequestration capacity of seagrass meadows—a critical assessment. Marine Pollution Bulletin.

[ref-77] Macreadie PI, Nielsen DA, Kelleway JJ, Atwood TB, Seymour JR, Petrou K, Connolly RM, Thomson ACG, Trevathan-Tackett SM, Ralph PJ (2017). Can we manage coastal ecosystems to sequester more blue carbon?. Frontiers in Ecology and the Environment.

[ref-78] Maderey-R LE, Torres-Ruata C (1990). Hidrografía e hidrometría, IV.6.1 (A). Atlas Nacional del México, II, Escala.

[ref-79] Marbà N, Arias-Ortiz A, Masqué P, Kendrick GA, Mazarrasa I, Bastyan GR, Garcia-Orellana J, Duarte CM (2015). Impact of seagrass loss and subsequent revegetation on carbon sequestration and stocks. Journal of Ecology.

[ref-80] Martin MA, Landis E, Bryson C, Lynaugh S, Mongeau A, Lutz S (2016). Blue carbon—nationally determined contributions inventory. appendix to: coastal blue carbon ecosystems. Opportunities for nationally determined contributions.

[ref-81] Mazarrasa I, Samper-Villarreal J, Serrano O, Lavery PS, Lovelock CE, Marbà N, Duarte CM, Cortés J (2018). Habitat characteristics provide insights of carbon storage in seagrass meadows. Marine Pollution Bulletin.

[ref-82] Mazda Y, Kobashi D, Okada S (2005). Tidal-Scale Hydrodynamics within Mangrove Swamps. Wetlands Ecology and Management.

[ref-83] Medina-Gómez I, Madden CJ, Herrera-Silveira J, Kjerfve B (2016). Response of Thalassia testudinum morphometry and distribution to environmental drivers in a pristine tropical lagoon. PLOS ONE.

[ref-84] Mendoza-Martínez JE (2017). Captura y emisiones de carbono en pastos marinos sometidos a perturbaciones naturales. Tesis de maestría, Centro de Investigación y de Estudios Avanzados del.

[ref-85] Moher D, Liberati A, Tetzaff J, Altman DG (2009a). Preferred reporting items for systematic reviews and meta-analyses: the PRISMA statement. Annals of Internal Medicine.

[ref-86] Moher D, Liberati A, Tetzaff J, Altman DG, The PRISMA Group (2009b). Prefered reporting items for systematic reviews and meta analyses: the prisma statement. PLOS Medicine.

[ref-87] Moodley L, Middelburg JJ, Herman PMJ, Soetaert K, De Lange GJ (2005). Oxygenation and organic-matter preservation in marine sediments: Direct experimental evidence from ancient organic carbon–rich deposits. Geology.

[ref-88] Ochoa-Gómez JG, Lluch-Cota SE, Rivera-Monroy VH, Lluch-Cota DB, Troyo-Diéguez E, Oechel W, Serviere-Zaragoza E (2019). Mangrove wetland productivity and carbon stocks in an arid zone of the Gulf of California (La Paz Bay, Mexico). Forest Ecology and Management.

[ref-89] Odum WE, Heald EJ (1972). Trophic analyses of an estuarine mangrove community. Bulletin of Marine Science.

[ref-90] Odum WE, McIvor CC, Smith TJ (1982). The ecology of the mangroves of south Florida: a community profile.

[ref-91] Ouyang X, Lee SY, Connolly RM (2017). The role of root decomposition in global mangrove and saltmarsh carbon budgets. Earth-Science Reviews.

[ref-92] Pendleton L, Donato DC, Murray BC, Crooks S, Jenkins WA, Sifleet S, Craft C, Fourqurean JW, Kauffman JB, Marbà N, Megonigal P, Pidgeon E, Herr D, Gordon D, Baldera A (2012). Estimating global blue carbon emissions from conversion and degradation of vegetated coastal ecosystems. PLOS ONE.

[ref-93] Phang VXH, Chou LM, Friess DA (2015). Ecosystem carbon stocks across a tropical intertidal habitat mosaic of mangrove forest, seagrass meadow, mudflat and sandbar. Earth Surface Processes and Landforms.

[ref-94] Pullin AS, Stewart GB (2006). Guidelines for systematic review in conservation and environmental management. Conservation Biology.

[ref-95] Rahman M, Khan NI, Hoque F, Hamed I (2015). Carbon stock in the Sundarbans mangrove forest: spatial variations in vegetation types and salinity zones. Wetlands Ecology and Management.

[ref-96] Ramírez-Ramírez J, Medina-Gómez I, Herrera-Silveira JA, Paz F, Wong J (2015). Diversity and C storage in a submerged aquatic vegetation community of a coastal lagoon environment. Estado Actual del Conocimiento del Ciclo del Carbono y sus Interacciones en México: Síntesis a 2014.

[ref-97] Robertson AI, Alongi DM (1995). Role of riverine mangrove forests in organic carbon export to the tropical coastal ocean: a preliminary mass balance for the Fly Delta (Papua New Guinea). Geo-Marine Letters.

[ref-98] Robertson AI, Alongi DM (2016). Massive turnover rates of fine root detrital carbon in tropical Australian mangroves. Oecologia.

[ref-99] Robertson AI, Daniel PA (1989). The influence of crabs on litter processing in high intertidal mangrove forests in tropical Australia. Oecologia.

[ref-100] Rodríguez-Zúñiga MT, Troche-Souza C, Vázquez-Lule AD, Márquez-Mendoza JD, Vázquez-Balderas B, Valderrama-Landeros L, Velázquez-Salazar S, Cruz-López MI, Ressl R, Uribe-Martínez A (2013). Manglares de México/Extensión, distribución y monitoreo.

[ref-101] Saintilan N, Rogers K, Mazumder D, Woodroffe C (2013). Allochthonous and autochthonous contributions to carbon accumulation and carbon store in southeastern Australian coastal wetlands. Estuarine, Coastal and Shelf Science.

[ref-102] Sanders CJ, Maher DT, Tait DR, Williams D, Holloway C, Sippo JZ, Santos IR (2016). Are global mangrove carbon stocks driven by rainfall?. Journal of Geophysical Research G: Biogeosciences.

[ref-103] Santini NS, Reef R, Lockington DA, Lovelock CE (2015). The use of fresh and saline water sources by the mangrove Avicennia marina. Hydrobiologia.

[ref-104] Santos DMC, Estrada GCD, Fernandez V, Estevam MRM, Souza BTDE, Soares MLG (2017). First assessment of carbon stock in the belowground biomass of brazilian mangroves. Anais da Academia Brasileira de Ciêcias.

[ref-105] Schile LM, Kauffman JB, Crooks S, Fourqurean JW, Glavan J, Megonigal JP (2017). Limits on carbon sequestration in arid blue carbon ecosystems. Ecological Applications.

[ref-106] Short FT, Short A, Novak CA, Finlayson CM, Milton GR, Prentice RC, Davidson NC (2016). Seagrasses. The Wetland Book: II: Distribution, Description and Conservation.

[ref-107] Shunula JP, Whittick A (1999b). Aspects of litter production in mangroves from unguja island, Zanzibar, Tanzania. Estuarine, Coastal and Shelf Science.

[ref-108] Spalding MD, Blasco F,  Field CD (1997). World mangrove atlas.

[ref-109] Sutton-Grier AE, Moore A (2016). Leveraging carbon services of coastal ecosystems for habitat protection and restoration. Coastal Management.

[ref-110] Teutli-Hernández C, Herrera-Silveira JA, Ceccon Y, Martínez-Garza C (2016). Estrategias de restauración de manglares de México: el caso Yucatán. Experiencias mexicanas en la restauración ecológica de ecosistemas.

[ref-111] Thorhaug A, Poulos HM, López-Portillo J, Ku TCW, Berlyn GP (2017). Seagrass blue carbon dynamics in the Gulf of Mexico: Stocks, losses from anthropogenic disturbance, and gains through seagrass restoration. Science of The Total Environment.

[ref-112] Thorhaug AL, Poulos HM, López-Portillo J, Barr J, Lara-Domínguez AL, Ku TC, Berlyn GP (2018). Gulf of Mexico estuarine blue carbon stock, extent and flux: Mangroves, marshes, and seagrasses: a North American hotspot. Science of The Total Environment.

[ref-113] Twilley RR, Lugo AE, Patterson-Zucca C (1986). Litter production and turnover in Basin Mangrove Forests in Southwest Florida. Ecology.

[ref-114] Twilley RR, Rivera-Monroy VH (2005). Developing performance measures of mangrove wetlands using simulation models of hydrology, nutrient biogeochemistry, and community dynamics. Journal of Coastal Research.

[ref-115] Twilley RR, Rovai AS, Riul P (2018). Coastal morphology explains global blue carbon distributions. Frontiers in Ecology and the Environment.

[ref-116] UNFCCC V (2015). United Nations Framework Convention on Climate Change. Adoption of the Paris agreement. I: proposal by the president (Draft Decision).

[ref-117] Valderrama-Landeros LH, Rodríguez-Zúñiga MT, Troche-Souza C, Velázquez-Salazar S, Villeda-Chávez E, Alcántara-Maya JA, Vázquez-Balderas B, Cruz-López MI, R Ressl (2017). Manglares de México: actualización y exploración de los datos del sistema de monitoreo 1970/1980–2015.

[ref-118] Wang M, Zhang J, Tu Z, Gao X, Wang W (2010). Maintenance of estuarine water quality by mangroves occurs during flood periods: a case study of a subtropical mangrove wetland. Marine Pollution Bulletin.

[ref-119] Waycott M, Duarte CM, Carruthers TJB, Orth RJ, Dennison WC, Olyarnik S, Calladine A, Fourqurean JW, Heck KL, Hughes AR (2009). Accelerating loss of seagrasses across the globe threatens coastal ecosystems. Proceedings of the National Academy of Sciences of the United States of America.

[ref-120] Wolanski E, Boorman LA, Chícharo L, Langlois-Saliou E, Lara R, Plater AJ, Uncles RJ, Zalewski M (2004). Ecohydrology as a new tool for sustainable management of estuaries and coastal waters. Wetlands Ecology and Management.

[ref-121] Woodroffe C, Robertson A, Alongi D (1992). Chapter 2: mangrove sediments and geomorphology. Tropical mangrove ecosystems.

[ref-122] Yulianto G, Soewardi K, Adrianto L (2016). The role of mangrove in support of coastal fisheries in Indramayu Regency, West Java, Indonesia. Aquaculture, Aquarium, Conservation & Legislation-International Journal of the Bioflux Society.

[ref-123] Zhang H, Yuan W, Dong W, Liu S (2014). Seasonal patterns of litterfall in forest ecosystem worldwide. Ecological Complexity.

