# Supplementary material for: Blue carbon of Mexico, carbon stocks and fluxes: a systematic review
Source: PeerJ. 2020 Apr 6;8:e8790. doi: 10.7717/peerj.8790 (PMC7144590; doi:10.7717/peerj.8790)
Supplement: Table S1 — Columns shows aboveground carbon average, belowground carbon average and total carbon average, with respective standard errors, minims and maxims for mangroves. [file peerj-08-8790-s003.docx]

|  | MANGROVES | | |
| --- | --- | --- | --- |
| Weather | **Above**  **C±S.E**  **(Min – Max)**  **(Mg C/ha)** | **Below**  **C±S.E**  **(Min – Max)**  **(Mg C/ha)** | **Average Total C±S.E**  **(Min – Max)**  **(Mg C/ha)** |
| Humid | *162.3 ± 14*  *(0.7 - 458)* | *512 ± 53*  *(23 - 1161)* | *674 ± 68*  *(4 - 1433)* |
| Sub-humid | *113.1 ± 9*  *(0.1 - 451)* | *407 ± 31*  *(9 - 2003)* | *520 ± 40*  *(0.6 - 2233)* |
| Arid | *77 ± 14*  *(1 - 253)* | *169 ± 19*  *(43 - 365)* | *246 ± 34*  *(25 - 442)* |
| Very arid | *59 ± 20*  *(3.8 - 123)* | *404 ± 83*  *(45 - 893)* | *464 ± 103*  *(31 - 893)* |

|  | MANGROVES | | |
| --- | --- | --- | --- |
| Ecology type of mangrove | **Above**  **C±S.E**  **(Min – Max)**  **(Mg C/ha)** | **Below**  **C±S.E**  **(Min – Max)**  **(Mg C/ha)** | **Average Total C±S.E**  **(Min – Max)**  **(Mg C/ha)** |
| Basin | *102.5 ± 13*  *(0.6 - 458)* | *396 ± 46*  *(43 - 2003)* | *498 ± 58*  *(163 - 2233)* |
| Dwarf | *14.4 ± 3*  *(0.1 - 68)* | *253 ± 23*  *(87 - 609)* | *267 ± 25*  *(39 - 618)* |
| Fringe | *149.4 ± 13*  *(1.9 - 405)* | *374 ± 41*  *(9 - 1047)* | *524 ± 53*  *(130 - 1098)* |
| Peten | *171.8 ± 38*  *(115 - 256)* | *932 ± 105*  *(850 - 1085)* | *1103 ± 143*  *(5 - 1201)* |
| Riverine | *156.4 ± 14*  *(6.5 - 451)* | *594 ± 55*  *(100 - 1225)* | *750 ± 69*  *(97 - 1601)* |
