# Supplementary material for: Blue carbon of Mexico, carbon stocks and fluxes: a systematic review
Source: PeerJ. 2020 Apr 6;8:e8790. doi: 10.7717/peerj.8790 (PMC7144590; doi:10.7717/peerj.8790)
Supplement: Supplemental Information 4 [file peerj-08-8790-s004.docx]

**Statement of Rationale and Contribution of the work.**

**Rationale and Contribution for a systematic review on Blue carbon stocks and ﬂuxes of Mexico**

**1. The rationale for conducting the systematic review**

It has been documented that blue carbon ecosystems are valuable in the social, economic and biological sense. They are special important to face the climate change in coasts globally, functions such as the carbon storage, are related to mitigation and adaptation strategies at the time they provide other ecosystem services, such as coastal protection, fisheries and biodiversity enhancement, and food security for many coastal communities. The study of blue carbon ecosystems is an emerging science which in addition to generate scientific knowledge, also constitute the base line for policies, conservation strategies and the forthcoming carbon market, then scientific community has been challenged by the potential users to address knowledge gaps and the evaluation of uncertainties required for policy and management proposes. The information generated is attractive to diverse users beyond the scientific community which include ONGs, private sector, governments, and intergovernmental bodies.

**2. The contribution that the systematic review makes to knowledge in light of previously published related reports.**

Our results demonstrate that Mexico present a high variability in blue carbon reservoirs according to diverse factors such as region, climate, and structure of the vegetation. The Yucatan Peninsula presented the largest blue carbon stock with low uncertainty values due to this region is well represented in scientific literature (number of studies), in addition to the mangroves and seagrasses extent as well as structural vegetation typology. However, this region is the most impacted for land use changes. At county level the information about C_org_ in seagrasses soil was scarce, and research gaps were evident in the carbon flux review. Systematic reviews at country level of the C_org_ stocks in blue carbon ecosystems around the world are unusual, and in case they do exist, only biomass component is considered. It is frequent to find general approaches at higher spatial resolution such as continents but the C_org_ stocks trends to be underestimated.
