# Supplementary material for: Blue carbon of Mexico, carbon stocks and fluxes: a systematic review
Source: PeerJ. 2020 Apr 6;8:e8790. doi: 10.7717/peerj.8790 (PMC7144590; doi:10.7717/peerj.8790)
Supplement: Supplemental Information 5 [file peerj-08-8790-s005.doc]

| **Section/topic** | **#** | **Checklist item** | **Reported on page #** |
| --- | --- | --- | --- |
| **TITLE** | | |  |
| Title | 1 | **Identify the report as a systematic review, meta-analysis, or both**.  Systematic review | 1 |
| **ABSTRACT** | | |  |
| Structured summary | 2 | **Provide a structured summary including, as applicable: background; objectives; data sources; study eligibility criteria, participants, and interventions; study appraisal and synthesis methods; results; limitations; conclusions and implications of key findings; systematic review registration number.**  **Brackground:**  “Mexico has more than 750,000 ha of mangroves and more than 400,000 ha of seagrasses. However, approximately 200,000 ha of mangroves and an unknown area of seagrass have been lost due to coastal development associated to urban, industrial and tourist purposes. Despite the importance of inventories of Blue carbon, there has not been reported an integrative synthesis for both ecosystems, which summarize the carbon stocks and mitigation value of the Greenhouse gases (GHG) or identify the information and data gaps”.  **Objective:**  “Evaluate Mexican blue carbon ecosystems”.  **Data sources and study eligibility criteria:**  “We used the data from 126 eligible studies for both ecosystems (n=1220)”.  **Synthesis methods:**  “We evaluated Mexican blue carbon ecosystems through a systematic review of the carbon stock using the standardized method of PRISMA”.  **Results and limitations:**  “Considering the official extension for both ecosystems in Mexico, mangrove contributions is 237.7 Tg C_org_ and seagrasses number correspond to 48.1 Tg C_org_. However, mangrove and seagrass areas are still at risk due to anthropogenic activities and gaps in the legal framework, which allow the land use change in mangrove and seagrasses areas, even thought our laws incorporate environmental compensation schemes to lessen the damage, it is not enough to reverse or absorb the impact over ecosystem services”.  **Conclusions and implications of key findings:**  “However, mangrove and seagrass areas are still at risk due to anthropogenic activities and gaps in the legal framework, which allow the land use change in mangrove and seagrasses areas, even thought our laws incorporate environmental compensation schemes to lessen the damage, it is not enough to reverse or absorb the impact over ecosystem services. The estimated emissions from the land use change under a conservative approach in mangroves of Mexico were around 24 Tg CO_2_e in the las 20 years. Therefore, the incorporation of the “blue carbon” initiative and the mechanism of carbon market into public policies is an attractive win-win opportunity which trend towards a suitable management for those ecosystems”.  **Participants and interventions:**  The authors Jorge Herrera-Silveira, generated the main idea of the study, Monica A. Pech-Cardenas, Andrea Camacho-Rico, Eunice Pech-Poot, Juan P. Caamal-Sosa, Claudia Teutli-Hernandez performed the literature review of the mangroves, Sara M. Morales-Ojeda, Juan E. Mendoza-Martinez and, Siuling Cinco-Castro performed the review of the seagrasses. Andrea Camacho-Rico, Sara M. Morales-Ojeda, Juan E. Mendoza-Martinez, Monica A. Pech-Cardenas, performed the figures and data management, and Jorge L. Montero-Muñoz were part of the statistical analysis. | 2 |
| **INTRODUCTION** | | |  |
| Rationale | 3 | **Describe the rationale for the review in the context of what is already known.**  “Among the coastal wet landscape, mangroves, seagrasses and salt marshes are known as blue carbon ecosystems; they **sequester** greenhouse gases **and store more** organic carbon over **the long term per unit area than terrestrial forests,** and they are now being recognized for their role in **mitigating climate change** (Pendleton et al., 2012). At an international level, it has been recognized that C_org_ sequestration and storage in vegetation and soil of blue carbon ecosystems could be a key component for mitigation strategies in the face of climate change.  Mexico has the largest extension of blue carbon ecosystems and is among the areas with the greatest coverage in the tropical and subtropical western hemisphere. Mexican Federal Government reports 755,555 ha of mangrove and 461,059 ha of seagrasses (Valderrama-Landeros et al., 2017; CONABIO, 2018)”.  “The mangrove national official regionalization scheme was proposed by a panel of scientists (CONABIO, 2009), but other classifications based on climate or ecological typology (Lugo & Snedaker, 1974) criteria have been useful to understand the variables related to the differences observed in carbon stocks, for example, among mangroves in wet and dry climates as well as among dwarf and hammock (peten) mangrove types, which could be of interest both for decision makers and for future research, at regional and local scales”.  “In the case of seagrasses, both the bathymetry gradient and coastal current velocities influence the water transparency, which is one of the key variables for seagrass development. The variability in water transparency is a factor related to the morphometric characteristics of the plants, which determine the aboveground C_org_ assessments of this ecosystem (Fourqurean et al., 2012a)”.  “In contrast, changes in the coverage of Mexico´s blue carbon ecosystems over time and their relation to changes in land use have been difficult to quantify due to the lack of long-term evaluation programs and robust data from C_org_ stocks, which in these ecosystems represent the main reservoir of C_org_  (Donato et al., 2011)”.  “However, despite Mexico´s commitment, and the availability of some but insufficient data has been collected, there has not been a complete synthesis of the mitigation value or the GHG emissions related to mangroves and seagrasses simultaneously”. | 3-5 |
| Objectives | 4 | **Provide an explicit statement of questions being addressed with reference to participants, interventions, comparisons, outcomes, and study design (PICOS).**  “The Mexican government is requesting information from mangrove and seagrass C_org_ stocks under different criteria that allow the development of conservation and restoration policies at different scales (INECC, 2018). In this context, it is necessary to consider the ecological and environmental scenarios in which mangroves and seagrasses develop to improve local, regional and national carbon inventories”. | 4 |
| **METHODS** | | |  |
| Protocol and registration | 5 | **Indicate if a review protocol exists, if and where it can be accessed (e.g., Web address), and, if available, provide registration information including registration number.**  There is no existing protocol that is yet available. | N/A |
| Eligibility criteria | 6 | Specify study characteristics (e.g., PICOS, length of follow-up) and report characteristics (e.g., years considered, language, publication status) used as criteria for eligibility, giving rationale.  A bibliographic review was carried out for carbon stokes and carbon fluxes in mangroves and seagrasses in Mexico, for which databases, scientific articles, thesis, publications of the Mexican Carbon Program (PMC), technical reports, congress reports, etc. were consulted. The data period was from 1987 to 2018. | 5 |
| Information sources | 7 | **Describe all information sources (e.g., databases with dates of coverage, contact with study authors to identify additional studies) in the search and date last searched.**  “This synthesis was carried out using information from published databases on C_org_ stocks in blue carbon ecosystems in Mexico (Herrera-Silveira et al., 2018a; Herrera-Silveira et al., 2018b)”.  Both mangroves and seagrasses databases have been validated, reviewed by experts and organizations of the federal government and civil society, and subjected to public consultation by the Mexican Carbon Program (<http://pmcarbono.org/pmc/>). | 5 |
| Search | 8 | **Present full electronic search strategy for at least one database, including any limits used, such that it could be repeated.**  “We use the Preferred Reporting Items for Systematic Reviews and Meta-Analyses (PRISMA) framework and protocols (Moher et al 2009)”. | 5 |
| Study selection | 9 | **State the process for selecting studies (i.e., screening, eligibility, included in systematic review, and, if applicable, included in the meta-analysis).**  “The studies included in the mangrove database used the sampling method in plots and for seagrasses the square method on transects. Studies that reported at least three plots as replicates were included”. | 5 |
| Data collection process | 10 | **Describe method of data extraction from reports (e.g., piloted forms, independently, in duplicate) and any processes for obtaining and confirming data from investigators.**  “The data were georeferenced; however, Once the coordinates were verified, we proceeded with standardization of the units of all the variables used”.  All the full-text accessed for elegibility contain data for organic carbon estimation.  “The authors of this synthesis have participated in assessments of the stocks and carbon fluxes in both ecosystems”. | 5 |
| Data items | 11 | **List and define all variables for which data were sought (e.g., PICOS, funding sources) and any assumptions and simplifications made.**  **Variables:**  *Above ground carbon*: Organic carbon of aboveground biomass as results by sum alive biomass and dead biomass in Mg ha^-1^.  *Below ground carbon:* Organic carbon of belowground carbon as results by the sum of the root and soil carbon in Mg ha^-1^.  The above and below ground biomass of all the studies was converted to organic carbon using the factor 0.45 for mangroves and 0.35 for seagrasses (Kauffman & Donato, 2012; Fourqurean et al., 2012a).  **Assumptions and simplifications**  “Corrections were made for inconsistencies in the location of the sites, derived from the different coordinate systems used in the studies”.  “In both ecosystems, the total soil C_org_ pool was standardized to a soil depth of 1 m, although there are reports in mangroves of depths of organic matter greater than 1 m (Caamal-Sosa et al., 2011; Adame & Fry, 2016; Ezcurra et al., 2016)”. | 5 |
| Risk of bias in individual studies | 12 | Describe methods used for assessing risk of bias of individual studies (including specification of whether this was done at the study or outcome level), and how this information is to be used in any data synthesis.  In order to have an evaluation of the uncertainty of the data in this synthesis, carbon stocks values of mangroves and seagrasses were grouping by region and nonparametric bootstrap confidence intervals were calculated using the method of adjusted bootstrap percentile (BCa whit B=10000) and bootstrap estimate of variance | 6 |
| Summary measures | 13 | State the principal summary measures (e.g., risk ratio, difference in means). | 6 |
| Synthesis of results | 14 | Describe the methods of handling data and combining results of studies, if done, including measures of consistency (e.g., I^2^) for each meta-analysis.  NA | NA |

| Risk of bias across studies | 15 | Specify any assessment of risk of bias that may affect the cumulative evidence (e.g., publication bias, selective reporting within studies). | NA |
| --- | --- | --- | --- |
| Additional analyses | 16 | Describe methods of additional analyses (e.g., sensitivity or subgroup analyses, meta-regression), if done, indicating which were pre-specified. | NA |
| **RESULTS** | | |  |
| Study selection | 17 | **Give numbers of studies screened, assessed for eligibility, and included in the review, with reasons for exclusions at each stage, ideally with a flow diagram.**    We uniquely identify 176 articles for seagrasses and mangroves, from them were screened out 50 for not containing useful information to calculate carbon stores, living as eligible and included 126 articles in this systematic review. | 5 |
| Study characteristics | 18 | **For each study, present characteristics for which data were extracted (e.g., study size, PICOS, follow-up period) and provide the citations.**  It should be noted that the studies included in the mangrove database used the sampling method in plots and for seagrasses the square method on transects. Studies that reported four to six plots as replicates were included.  Mangroves:  **Félix-Pico, E., Holguín-Quiñones, O., Hernández-Herrera, A. & Flores-Verdugo, F. 2006. Producción primaria de los mangles del Estero El Conchalito en Bahía de La Paz (Baja California Sur, México). *Ciencias marinas* *32*(1a): 53-63.** It was chosen because contains carbon fluxes data due to litterfall in mangroves.  **Valdés-Velarde, E., Valdez-Hernández, J., Ordaz-Chaparro, V., Gallardo-Lancho, J., Pérez-Nieto, J. & Ayala-Sánchez, C. 2011. Evaluación del carbono orgánico en suelos de los manglares de Nayarit. *Revista mexicana de ciencias forestales* 2(8): 47-58.** It was chosen because contains below ground carbon stock data of mangroves.  **Vazques-Lule, A., Couturier, S., Schmidt, M., Colditz, R., Silván-Cárdenas, J. & Llamas-Barba, R. 2012. The estimation of aerial biomass and structural parameters of mangroves in Laguna Pom Atasta, Campeche and Laguna Agua Brava, Nayarit, Mexico, using ALOS PALSAR radar images. *XV^th^ Simposio de la Sociedad Latinoamericana de Percepción Remota y Sistemas de Información Espacial (SELPER)*. Cayenne, Guyana Francesa, 23-27.** It was chosen because contains above ground carbon stock data of mangroves.  **Arreola-Lizárraga, J. A., Flores-Verdugo, F. J., & Ortega-Rubio, A. 2004. Structure and litterfall of an arid mangrove stand on the Gulf of California, Mexico. *Aquatic botany* *79*(2): 137-143.** It was chosen because contains carbon fluxes data due to litterfall in mangroves.  **Flores-Verdugo, F. J., Day Jr, J. W. & Briseño-Dueñas, R. 1987. Structure, litter fall, decomposition, and detritus dynamics of mangroves in a Mexican coastal lagoon with an ephemeral inlet. *Marine Ecology Progress Series* *35*: 83-90.** It was chosen because contains carbon fluxes data for mangroves.  **Colado-Amador, C. 2015. Estimación de la biomasa en manglares Áridos en la bahía del Tobari y estero El Sargento, en Sonora, México. En: *Simposio Internacional del Carbono en México 2015*, Programa Mexicano del Carbono. Tabasco, México. 61 pp.** It was chosen because contains mangrove biomass data wich can be converted in carbon with factors.  **Robles-Zazueta, C., Rodríguez, J., Castellanos, A., López, E., Garatuza-Payan, J., Meling-López, A. & Watts, C. 2015. Reservorios de carbono en suelos de manglar bajo diferentes antropogénicas en el Golfo de California. En: *Simposio Internacional del Carbono en México 2015*, Programa Mexicano del Carbono. Tabasco, México. 106 pp.** It was chosen because contains below ground carbon stock data of mangroves.  **Rivera-Aldaco, Y., Nájera-González, O., Murray-Núñez, R. 2013. Reserva de carbono en la biomasa aérea de manglar. En: *Simposio Internacional del Carbono en Mexico 2015*, Programa Mexicano del Carbono. Monterrey, México. 292-298.** It was chosen because contains biomass and above ground carbon stock data of mangroves.  **Ezcurra, P., Ezcurra, E., Garcillán, P., Costa, M. & Aburto-Oropeza, O. 2016. Coastal landforms and accumulation of mangrove peat increase carbon sequestration and storage. *Proceedings of the National Academy of Sciences* *113*(16): 4404-4409.** It was chosen because contains below ground carbon stock data calculated by a standarized metodology.  **Méndez-Linares, A., López-Portillo, J., Hernández-Santana, J., Pérez, M. & Orozco, O. 2007. The mangrove communities in the Arroyo Seco deltaic fan, Jalisco, Mexico, and their relation with the geomorphic and physical–geographic zonation. *Catena* *70* (2): 127-142.** It was chosen because contains mangrove forestry structure data.  **Estrada-Duran, G. 2000. Estructura y producción de hojarasca del bosque de manglar de estero El Salado, Puerto Vallarta, Jalisco, México (Tesis de licenciatura). Universidad de Guadalajara, Jalisco, 35 pp.** It was chosen because contains forestry structure data and carbon fluxes data due to litterfall in mangroves.  **Téllez-García, C. & Valdez-Hernández, J. 2012. Caracterización estructural del manglar en el estero Palo Verde, laguna de Cuyutlán, Colima. *Revista Chapingo serie ciencias forestales y del ambiente* *18*(3): 395-408.** It was chosen because contains mangrove structure data.  **Jiménez-Quiros, M.C. & Gonzalez, F. 1996. Análisis de la estructura del manglar de la laguna Juluapan, Colima, México. INP. SEMARNAP. *Ciencia Pesquera 12*: 76-84.** It was chosen because contains mangrove structure data.  **Estrada-Durán, G., Cupul-Magaña, F. & Cupul-Magaña, A. 2001. Aspectos de la estructura y producción de hojarasca del bosque de manglar del estero El Salado, Puerto Vallarta, Jalisco. *Ciencia y Mar 5*(15): 3-12.** It was chosen because contains forestry structure data and carbon fluxes data due to litterfall in mangroves.  **Mendoza-Morales, A.J., González-Sansón, G. & Aguilar-Betancourt, C. 2016. Producción espacial y temporal de hojarasca del manglar en la laguna Barra de Navidad, Jalisco, México. *Revista de Biología Tropical* 64(1): 259-273.** It was chosen because contains forestry structure data and carbon fluxes data due to litterfall in mangroves.  **Orihuela-Belmonte, D.E., Tovilla-Hernández, C., Vester, H.F., & Álvarez-Legorreta, T. 2004. Flujo de materia en un manglar de la costa de Chiapas, México. *Madera y Bosques* *10*(Es2): 45-61.** It was chosen because contains forestry structure data and carbon fluxes data due to litterfall in mangroves.  **Colón-Alarcón, M. 2014. Estructura arbórea de manglares en Tecoanapa, Guerrero, México (Tesis de licenciatura). Universidad Autónoma Agraria Antonio Narro. Coahuila, México. 53 pp.** It was chosen because contains mangrove forestry structure data useful to calculate above ground carbon stocks.  **Tovilla-Hernández, C., Ovalle-Estrada, F., De la Presa-Pérez, J., Gonzalez-Castillo, D., De la Cruz-Montes, E. & De la Cruz-Montes, G. 2012. Inventario y monitoreo del estado actual de los bosques de manglar de Chiapas y Oaxaca, México. CONABIO Proyecto FN005.** It was chosen because contains mangrove forestry structure data useful to calculate above ground carbon stocks. And the size of the study.  **Adame, M. F., Santini, N. S., Tovilla, C., Vázquez-Lule, A. & Castro, L. 2015. Carbon stocks and soil sequestration rates of riverine mangroves and freshwater wetlands. *Biogeosciences Discussions* 12(2): 015-1045.** It was chosen because contains below ground carbon stock data calculated by a standarized metodology.  **Tovilla-Hernández, C., De la Presa-Pérez, J.C., Ovalle-Estrada, F., De la Cruz-Montes, G. & De la Cruz-Montes, E. 2010. Estructura de los bosques de manglar de *Avicennia bicolor* (Standley 1923) en la costa de Chiapas, México. En: Primer Congreso Mexicano de Ecosistemas. Yucatán, México. 83 pp.** It was chosen because contains mangrove forestry structure data useful to calculate above ground carbon stocks.  **Tovilla-Hernández, C. & Romero-Berny, E. 2012. Diagnóstico estructural de los manglares de Chiapas y Oaxaca. En: Chiappa-Carrará, X., Sánchez-Martínez, A. & Brito-Pérez, R (eds.). *Recursos acuáticos costeros del sureste*, pp. 257-279. UNAM / CONCITEY. Yucatán, México.**  It was chosen because contains mangrove forestry structure data.  **Tovilla-Hernández, C., & Romero-Berny, E. I. 2012. Diagnóstico estructural de los manglares de Chiapas y Oaxaca. Recursos acuáticos costeros del Sureste 1: 257-279.** It was chosen because contains mangrove forestry structure data.  **Bejarano, M., Herrera-Silveira, J. & Carillo, O. 2015. Estimación de almacenes de carbono en manglares: una adaptación para la costa de Chiapas. Pronatura Sur A.C. 2015. Potencial REDD+ del Corredor de Manglares Chiapas-Oaxaca. 44.** It was chosen because contains total carbon stock data calculated by a standarized metodology.  **Torres-Torres, R., Ramírez, A., Najib, F. 2013. Los manglares de Veracruz: Potencial de captura de carbono. Informe técnico del proceso de medición de carbono en 33 parcelas de manglar del sistema lagunar de Alvarado, Veracruz, México. PRONATURA. Xalapa, Veracruz. 119 pp.** It was chosen because contains total carbon stock data.  **Kauffman, J.B., Hernández-Trejo, H., Jesus-García, M.D., Heider, C. & Contreras, W.M. 2016. Carbon stocks of mangroves and losses arising from their conversion to cattle pastures in the Pantanos de Centla, Mexico. *Wetlands Ecology and Management* *24*(2): 203-216.** It was chosen because contains total carbon stock data calculated by a standarized metodology.  **Aké-Castillo, J. A., Vázquez, G. & López-Portillo, J. 2006. Litterfall and decomposition of Rhizophora mangle L. in a coastal lagoon in the southern Gulf of Mexico. *Hydrobiologia* *559*(1): 101-111.** It was chosen because contains data about carbon fluxes by litterfall.  **Utrera-López, M.E. & Moreno-Casasola, P. 2008. Mangrove litter dynamics in la mancha lagoon, Veracruz, México. *Wetlands Ecology and Management* *16*(1): 11-22.** It was chosen because contains data about carbon fluxes by litterfall and also for the historical data.  **Calva, L.G. & Torres-Alvarado, R. 2000. Distribución de carbohidratos, carbono y nitrógeno orgánico en sedimentos de tres lagunas costeras del Golfo de México. *Hidrobiológica* *10*(2): 101-114**. t was chosen because contains below ground carbon stock data of mangroves.  **Moreno-Cáliz, E., Guerrero-Peña, A., Gutiérrez-Castorena, M.C., Ortiz-Solorio, C. & Palma-López, D. 2002. Los manglares de Tabasco, una reserva natural de carbono. *Madera y Bosques* Número especial: 115-128.** It was chosen because contains above ground carbon stock data of mangroves.  **Herrera-Silveira, J.A., Caamal-Sosa J.P., Carrillo B.L., Pech P.E., Pech C. M., Erosa A.J., Zenteno D.K., Gamboa S.S. 2017. Medición de reservas de carbono y tasas de secuestración de carbono en la Reserva Privada Moon Palace. Informe Final. Programa Mexicano del Carbono-CINVESTAV-IPN 28 pp.** It was chosen because contains total carbon stock data calculated by a standarized metodology.  **Jorge A. Herrera-Silveira, Andrea Camacho Rico, Siuling Cinco Castro, Juan Caamal Sosa, Juan Mendoza Martínez, Ana Borges Miss. 2017. Evaluación de los almacenes de carbono en sitios piloto. Informe Técnico. Proyecto *CSP-2016-057* Programa de las Naciones Unidas para el Desarrollo. Programa Mexicano del Carbono. Centro de Investigación y de estudios avanzados del Instituto Politécnico Nacional. México. 139 pp.** It was chosen because contains total carbon stock data calculated by a standarized metodology.  **Marín-Muñiz, J.L., Hernández-Alarcón, M.E. & Casasola-Barceló, P.M. 2011. Secuestro de carbono en suelos humedales costeros de agua dulce en Veracruz. *Tropical and Subtropical Agroecosystems* *13*(3): 365-372.** It was chosen because contains below ground carbon stock data of mangroves.  **Méndez‐Alonzo, R., López‐Portillo, J. & Rivera‐Monroy, V.H. 2008. Latitudinal variation in leaf and tree traits of the mangrove *Avicennia germinans* (Avicenniaceae) in the central region of the Gulf of Mexico. *Biotropica* 40(4): 449-456.** It was chosen because contains data about carbon fluxes by litterfall.  **Agraz-Hernández, C., Garcia-Zaragoza, C., Iriarte-Vivar, S., Flores-Verdugo, F. J. & Moreno- Casasola, P. 2011. Forest structure, productivity and species phenology of mangroves in the La Mancha lagoon in the Atlantic coast of Mexico. *Wetlands Ecology and Management* *19*(3): 273-293.** It was chosen because contains mangrove biomass data wich can be converted in carbon with factors.  **Muñoz, A. D. J. B., Pérez, G. O., & Mencio, P. R. 2006. Características estructurales y usos del manglar en el ejido Cerro de Tumilco, Tuxpan, Veracruz. México. *Revista Científica UDO Agrícola* 6(1): 114-120.** It was chosen because contains mangrove biomass data wich can be converted in carbon with factors.  **Basáñez-Muñoz, A.J., Cruz-Lucas, M.A., Domínguez-Barradas, C., González-Gándara, C., Serrano-Solis, A. & Hernández-Azuara, A. 2008. Estructura y producción de *Conocarpus erectus* L. en el Sitio Ramsar “Manglares y Humedales de Tuxpan”, Veracruz, México. *UDO Agrícola* *8*(1): 78-87.** It was chosen because contains mangrove forestry structure and litterfall data.  **López-Portillo, J. A. 2012. Programa regional para la caracterización y el monitoreo de ecosistemas de manglar del Golfo de México y Caribe Mexicano: inicio de una red multi-institucional. Veracruz. Instituto de Ecología AC Informe final SNIB-CONABIO, proyecto No. FN007. México.** It was chosen because contains mangrove biomass data wich can be converted in carbon with factors. And by the size of the study  **Domínguez-Domínguez, M., Zavala-Cruz, J. & Martínez-Zurimendi, P. 2011. Manejo forestal sustentable de los manglares de Tabasco. Secretaria de Recursos Naturales y Protección Ambiental. Colegio de Postgraduados. Villahermosa, Tabasco. México. 137 pp.** It was chosen because contains mangrove biomass data wich can be converted in carbon with factors.  **Lara-Domínguez, A.L., Day, J.W., Sainz-Hernández, E., González, A. & Marín, R. 2010. Estructura y productividad de los manglares asocioados a las lagunas La Mancha y el Ostión, Veracruz, México. En: Primer Congreso Mexicano de Ecosistemas. Yucatán, México. 78 p.** It was chosen because contains mangrove forestry structure and litterfall data.  **Anacleto-Rosas, A.D., Hernández-Trejo, H. & Jesús-Garcia, M.C. 2010. Estructura del mangle botoncillo *Conocarpus erectus* L. en el Ejido la Victoria, Centla, Tabasco. En: Primer congreso mexicano de ecosistemas de manglar. Yucatán, México. 145 pp.** It was chosen because contains mangrove forestry structure data.  **Corella-Justavino, F., Valdez-Hernández, J.I., Cetina-Alcalá, V.M., González-Cossio, F., Trinidad-Santos, A. & Aguirre-Rivera, J.R. 2001. Estructura forestal de un bosque de mangles en el noreste del estado de Tabasco, México. *Ciencia forestal en México* *26*(90): 73-102.** It was chosen because contains mangrove forestry structure data.  **Vázquez-Lule, A., Couturier, S., Schmidt, M., Colditz, R., Silván-Cárdenas, J. & Llamas-Barba, R. 2012. The estimation of aerial biomass and structural parameters of mangroves in Laguna Pom Atasta, Campeche and Laguna Agua Brava, Nayarit, Mexico, using ALOS PALSAR radar images. *XVth Simposio de la Sociedad Latinoamericana de Percepción Remota y Sistemas de Información Espacial (SELPER)*, Cayenne, Guyana Francesa, 23-27.** It was chosen because contains mangrove biomass data wich can be converted in carbon with factors.  **Day, J. W., Coronado-Molina, C., Vera-Herrera, F. R., Twilley, R., Rivera-Monroy, V. H., Alvarez-Guillen, H., Day, R. & Conner, W. 1996. A 7 year record of above-ground net primary production in a southeastern Mexican mangrove forest. *Aquatic Botany* *55*(1): 39-60.** It was chosen because contains data about carbon fluxes by litterfall and also for the historical data.  **Adame, M. F., Zaldívar‐Jiménez, A., Teutli, C., Caamal, J. P., Andueza, M. T., López‐Adame, H., Cano, R., Hernández-Arana, H., Torres-Lara, R. & Herrera‐Silveira, J. A. 2013. Drivers of mangrove litterfall within a karstic region affected by frequent hurricanes. *Biotropica* *45*(2): 147-154.** It was chosen because contains total carbon stock data calculated by a standarized metodology.  **De Jesús-Navarrete, A., & Oliva-Rivera, J.J. (2014). Litter production of *Rhizophora mangle* at Bacalar Chico, Southern Quintana Roo, Mexico. *Ecosistemas y Recursos Agropecuarios* *18*(36): 79-86.** It was chosen because contains data about carbon fluxes by litterfall.  **Lara-Domínguez, A. L., Day, J. W., Villalobos-Zapata, G., Twilley, R. R., Álvarez-Guillén, H. & Yáñez-Arancibia, A. 2005. Structure of a unique inland mangrove forest assemblage in fossil lagoons on the Caribbean Coast of Mexico. *Wetlands Ecology and Management* 13(2): 111-122.** It was chosen because contains mangrove forestry structure data.  **Zaldívar-Jiménez, A., Herrera-Silveira, J., Coronado-Molina, C. & Alonzo-Parra, D. 2004. Estructura y productividad de los manglares en la reserva de biosfera Ría Celestún, Yucatán, México. *Madera y Bosques* *10*(2): 23-35.** It was chosen because contains mangrove forestry structure data and carbon fluxes data.  **Cerón, R.M., Cerón, J.G., Guerra, J.J., Zavala, J.C., Amador, L.E., Endañu, E. & Moreno, G. 2010. Estimación de la tasa de captura de carbono en ecosistemas de manglar asociados al área naural protegida Laguna de Términos. En: Congreso Internacional de Químicos Farmacéuticos Biólogos 2010. 24-25 agosto. Monterrey, NL.** It was chosen because contains below ground carbon stock data of mangroves.  **Cerón-Bretón, J. G., Cerón-Bretón, R. M., Rangel-Marrón, M., Muriel-Garcia, M., Cordova-Quiroz, A. V. & Estrella-Cahuich, A. 2011. Determination of carbon sequestration rate in soil of a mangrove forest in Campeche, Mexico. *WSEAS Transactions on Environment and Development* *7*(2): 55-64.** It was chosen because contains below ground carbon stock data of mangroves.  **Moreno-May G., Cerón-Breton R.M., Cerón-Breton J.G., Endañu-Huerta E., Amador-del Angel L.E., Guerra Santos J.J. 2010. Organic matter content in mangrove soils with associations of rhizophora mangle and laguncularia racemosa.Primer Congreso Nacional de Ingenieria Química Universidad Autónoma del Carmen, México.** It was chosen because contains useful data to calculate carbon below ground of mangroves.  **Adame, M. F., Kauffman, J. B., Medina, I., Gamboa, J. N., Torres, O., Caamal, J. P., Reza, M. & Herrera-Silveira, J.A. 2013. Carbon stocks of tropical coastal wetlands within the karstic landscape of the Mexican Caribbean. *PLoS One 8*(2): 1-13.** It was chosen because contains total carbon stock data calculated by a standarized metodology.  **Cerón-Bretón, R. M., Cerón-Bretón, J. G., Sánchez-Junco, R. C., Damián-Hernández, D. L., Guerra-Santos, J. J., Muriel-Garcia, M. & Córdova-Quiroz, A. V. 2011. Evaluation of carbon sequestration potential in mangrove forest at three estuarine sites in Campeche Mexico. *International Journal of energy and environment* *5*(4): 487-494.** It was chosen because contains below ground carbon stock data of mangroves.  **Agraz-Hernández, C. M., Osti-Sáenz, J., Chan-Keb, C. A., Chan-Canul, E., Gómez-Ramírez, D., Requena-Pavón, G. & Reyes-Castellanos, J.E. 2012. Programa regional para la caracterización y el monitoreo de ecosistemas de manglar del Golfo de México y Caribe Mexicano: Campeche. Universidad Autónoma de Campeche. Centro de Ecología Pesquerías y Oceanografía del Golfo de México. Informe Final SNIB-CONABIO. Proyecto FN010. México, D.F.** It was chosen because contains mangrove forestry structure data.  **Castillo-Domínguez, S. 2013. Determinación de unidades ambientales del ecosistema de manglar como instrumento de gestión en la Laguna de Términos, Campeche (Tesis de Maestría). Universidad Autónoma de Campeche. Campeche, Mex.** It was chosen because contains mangrove forestry structure data.  **Damián-Hernández, D.L., Sánchez-Junco, R., Arencibia-Carballo, G., Cerón-Bretón, R.M., Cerón-Bretón, J.G. 2010. Carbono y nitrógeno de suelos con cobertura de *Rhizophora mangle*, *Avicennia germinan*s, *Laguncularia racemosa* y *Conocarpus erectus* de la Península de Atasta, Campeche. En: Primer Congreso Mexicano de Ecosistemas. Yucatán, México. 117 pp.** It was chosen because contains below ground carbon stock data of mangroves.  **Herrera-Silveira, J.A., Teutli-Hernández, C., Zaldívar-Jiménez, A., Pérez-Ceballos, R., Cortés-Balán, O., Osorio-Moreno, I., Ramirez-Ramirez, J., Caamal-Sosa., J., Andueza-Briceño, M.T., Torres, R. & Hernández-Aranda, H. 2011. Programa regional para la caracterización y el monitoreo de ecosistema de manglar del Golfo de México y el Caribe mexicano: Inicio de una red multi-institucional: Península de Yucatán. Informe final. CINVESTAV-ECOPEY/CONABIO, proyecto FB1307-N009/08. Septiembre 2013.** It was chosen because contains mangrove biomass data wich can be converted in carbon with factors.  **Conde-Medina, K. P. 2011. Conservación y vulnerabilidad actual y a futuro del ecosistema de mangle que bordea al Rio Icahao en Champotón, Campeche, México (Tesis de Licenciatura). Campeche, México.** It was chosen because contains mangrove forestry structure data.  **Caamal-Sosa, J.P. 2012. Almacenes de carbono en diferentes tipos ecológicos de manglares en un escenario cárstico (Tesis de Maestría). Departamento de Recursos del Mar, Centro de Investigación y Estudios Avanzados del IPN Unidad Mérida. Mérida, Yucatán. 55 pp.** It was chosen because contains total carbon stock data calculated by a standarized metodology.  **Gutiérrez-Mendoza, J. 2014. El Servicio ecosistémico de almacenes de carbono en manglares del tipo ecológico Chaparro en un escenario cárstico (Tesis de Maestría). Departamento de Recursos del Mar, Centro de Investigación y Estudios Avanzados del IPN Unidad Mérida. Mérida, Yucatán. 80 pp.** It was chosen because contains total carbon stock data calculated by a standarized metodology.  **Camacho-Rico, A. 2018. Factores determinantes en la dinámica de intercambio de carbono y nutrientes entre el manglar y la laguna costera de Celestún (Tesis de Doctorado). Centro de Investigación y de Estudios Avanzados del IPN Unidad Mérida. Mérida, Yucatán, México.** It was chosen because contains total carbon stock data calculated by a standarized metodology. Also contains carbon fluxes data in mangroves.  **Coronado-Molina, C., Álvarez-Guillen, H., Day, J. W., Reyes, E., Pérez, B. C., Vera-Herrera, F. & Twilley, R. 2012. Litterfall dynamics in carbonate and deltaic mangrove ecosystems in the Gulf of Mexico. *Wetlands Ecology and Management* *20*(2): 123-136.** It was chosen because contains data about carbon fluxes by litterfall.  **Zaldívar-Jiménez, A., Herrera-Silveira, J.A., Teutli-Hernández, C., Pech, D., Hernández-Saavedra, R., Caamal, J., Andueza, T., Pérez-Ceballos, R. & Coronado-Molina, C. 2010. Variabilidad de largo plazo en la caída de hojarasca del manglar en una laguna costera cárstica (Península de Yucatán): Período 1999-2006. En: Primer Congreso Mexicano de Ecosistemas. Yucatán, México. 81 pp.** It was chosen because contains data about carbon fluxes by litterfall and also for the historical data.  **López-Adame, H., Hernández-Arana, H., Zaldívar-Jiménez, A. & Herrera-Silveira, J.A. 2010. Variación en la estructura, salinidad intersticial y producción de hojarasca en manglares de la bahía de Chetumal, México. En: Primer Congreso Mexicano de Ecosistemas. Yucatán, México. 153 p.** It was chosen because contains mangrove forestry structure data and carbon fluxes data.  **Herrera-Silveira, J.A., J. Caamal Sosa, J, Mendoza Martínez, K. Centeno Díaz, H. Us Balam, E. Pech, J. Erosa Angulo, y O. Pérez Martínez. 2018. EVALUACIÓN DE CARBONO AZUL DE LOS MANGLARES DE COZUMEL COMO ESTRATEGIA DE FACTIBILIDAD PARA EVITAR, REDUCIR Y/O MITIGAR EMISIONES DE GASES DE EFECTO INVERNADERO. Informe Técnico Final. PMC-CINVESTAV-CEMDA.** It was chosen because contains total carbon stock data calculated by a standarized metodology.  **Cerón-Bretón, J. G., Cerón-Bretón, R. M., Guerra-Santos, J. J. & Córdova-Quiroz, A. V. 2014. Estimation of regional carbon storage potential in mangrove soils on Carmen Island, Campeche, Mexico. En: Morgado, C. & Esteves, V. 2014. CO_2_ Sequestration and Valorization. inTech. 472 pp.** It was chosen because contains below ground carbon stock data of mangroves.  **Barreiro-Güemes, M. 1999. Aporte de hojarasca y renovación foliar del manglar en un sistema estuarino del Sureste de México. *Revista de Biología Tropical* 47(4): 729-737.** It was chosen because contains data about carbon fluxes by litterfall,  **Thorhaug, A. L., Poulos, H. M., López-Portillo, J., Barr, J., Lara-Domínguez, A. L., Ku, T. C. & Berlyn, G. P. 2018. Gulf of Mexico estuarine blue carbon stock, extent and flux: Mangroves, marshes, and seagrasses: A North American hotspot. *Science of the Total Environment*, 653: 1253-1261.** It was chosen because contains total carbon stock data calculated by a standarized metodology.  Seagrasses:  **Herrera-Silveira JA., Mendoza-Martínez J., Morales-Ojeda S., Camacho-Rico A., Medina-Gómez I., Ramírez-Ramírez I., López-Herrera M., Pech-Poot E., Pérez-Martínez O., Pech-Cárdenas M, Cota-Lucero T., Teutli-Hernández C. 2018b. Database of carbon stocks in seagrasses of Mexico. *Elementos para Políticas Públicas* 2(1): 45-52.**  Chosen because contains data about above and below ground carbon stocks in seagrasses.  **Herrera-Silveira J., Camacho Rico, A., Cinco Castro S., Caamal Sosa J., Mendoza Martínez J., Borges Miss A. 2017. Evaluación de los almacenes de carbono en sitios piloto. Informe Técnico. Proyecto CSP-2016-057 Programa de las Naciones Unidas para el Desarrollo. Programa Mexicano del Carbono. Centro de Investigación y de estudios avanzados del Instituto Politécnico Nacional. México. 139 pp.** Chosen because contains data about above and below ground carbon stocks in seagrasses calculated with a standarized metodology.  **Sinicrope Talley, T., Dayton, K. & Ibarra-Obando, S. 2000. Tidal flat macrofaunal communities and their associated environments in estuaries of southern california and northern Baja California, Mexico. *Estuaries* 23 (1): 97-114.** Chosen because contains data about seagrass biomass.  **Quiroz-Vázquez, P., Ibarra-Obando, S. and Meling-López, A. 2005. Composition of the epifaunal community associated with the seagrass *Zostera marina* in San Quintin Bay Baja California. *Bulletin of Southern California Academy of Sciences* 104 (2).** Chosen because contains data about seagrass biomass.  **Jorgensen, P., Ibarra-Obando, S. & Carriquiry, J. 2007. Top-down and bottom-up stabilizing mechanisms in seagrass meadows differentially affected by coastal upwelling. *Marine Ecology Progress Series* 333: 81-93.** Chosen because contains data about seagrass biomass.  **Hernández Sánchez, G. 2014. Identificación de las fuentes de nitrógeno para pastos marinos y macroalgas en bahía de San Quintín bajo condiciones de surgencias y no surgencia mediante análisis de isótopos estables de nitrógeno. Tesis de Maestría CICESE. Baja California, México. 88 pp.**  **Jorgensen, P. 2006. Control de *Zostera marina* por consumidores y recursos en praderas sumergidas bajo diferentes regímenes de fertilización natural. Tesis Doctoral. CICESE. Baja California, México. 190 pp.** Chosen because contains data about seagrass biomass.  **Villegas Martínez. K. 2015. Cambios estequiométricos de las hojas del pasto marino *Zostera marina* en Bahía San Quintín: efectos sobre los mesoherbívoros. Tesis de Maestría. CICESE. Baja California, México. 98 pp.** Chosen because contains data about seagrass biomass.  **Kantún Manzano, C. y Herrera-Silveira, J.A. 2014. Influencia de las descargas de agua subterránea en los almacenes de carbono azul en dos praderas de pastos marinos en Yucatán. En: Paz, F., J. Wong. (editores). 2015. Estado Actual del Conocimiento del Ciclo del Carbono y sus Interacciones en México: Síntesis a 2014. Serie Síntesis Nacionales. Programa Mexicano del Carbono en colaboración con el Centro de Investigación y Estudios Avanzados del Instituto Politécnico Nacional, Unidad Mérida y el Centro de Investigación y Asistencia en Tecnología y Diseño del Estado de Jalisco. Texcoco, Estado de México, México. ISBN: 978-607-96490-2-9. 642 p. C**hosen because contains data about above and below ground carbon stocks in seagrasses calculated with a standarized metodology.  **Mendoza-Martínez, J.E. 2017. Captura y Emisiones de Carbono en pastos marinos sometidos a perturbaciones naturales. Tesis de Maestría. Centro de Investigación y de estudios Avanzados del Instituto Politécnico Nacional CINVESTAV. Mérida, Yucatan. 98 pp.** Chosen because contains data about above and below ground carbon stocks in seagrasses calculated with a standarized metodology.  **May-Kú, M. A., Ardisson, P. L., and Ordónez-López, U. 2010. Morphological variation of *Thalassia testudinum* in two shallow coastal environments from the southeastern Gulf of Mexico. *Botanica Marina* 53(5): 449-455.** Chosen because contains data about seagrass biomass.  **Calva-Benítez, L. G., y Torres-Alvarado, R. 2011. Carbono orgánico y características texturales de sedimentos en áreas del pasto marino *Thalassia testudinum* en ecosistemas costeros del sureste del golfo de México. *Universidad y ciencia* 27(2): 133-144.** Chosen because contains data about carbon stocks in seagrasses soils.  **Ordóñez-López, U., y García-Hernández, V. D. 2005. Ictiofauna juvenil asociada a *Thalassia testudinum* en laguna Yalahau, Quintana Roo. *Hidrobiologica* 15(2): 195-204.** Chosen because contains data about seagrass biomass.  **Enríquez, S., and Pantoja-Reyes, N. I. 2005. Form-function analysis of the effect of canopy morphology on leaf self-shading in the seagrass *Thalassia testudinum*. *Oecologia* 145(2): 234-242.** Chosen because contains data about seagrass biomass.  **Olivera-Gómez, L. D., and Mellink, E. 2013. Aquatic Macrophytes Within a Mesohaline Bay, Sanctuary for Manatees (*Trichehus manatus*), on the Caribbean Coast of Mexico. *The Southwestern Naturalist* 58(2): 216-222.** Chosen because contains data about seagrass biomass.  **Gutiérrez-Aguirre, M. A., de la Fuente-Betancourt, M. G., & Cervantes-Martínez, A. 2000. Biomasa y densidad de dos especies de pastos marinos en el sur de Quintana Roo, México. Revista de Biología Tropical, 48(2-3), 313-316.** Chosen because contains data about seagrass biomass.  **Pantoja-Reyes, N. 2003. Evaluación de la importancia de la luz como factor regulador de la productividad foliar del pasto marino *T. testudinum* en la laguna arrecifal de Puerto Morelos. Tesis de Maestría. Universidad Nacional Autónoma de México. 99 pp.** Chosen because contains data about seagrass biomass.  **Duarte, C. M., Merino, M., Agawin, N. S., Uri, J., Fortes, M. D., Gallegos, M. E., & Hemminga, M. A. 1998. Root production and belowground seagrass biomass. *Marine Ecology Progress Series* 171: 97-108.** Chosen because contains data about below ground prodcutuctivity in seagrasses.  **Flores-Verdugo, F. J., Day, J. W., Mee, L., & Briseño-Dueñas, R. 1988. Phytoplankton production and seasonal biomass variation of seagrass, Ruppia maritima L., in a tropical Mexican lagoon with an ephemeral inlet. *Estuaries and Coasts*11(1): 51-56.** Chosen because contains data about seagrass biomass variation.  **van Tussenbroek, B. I. 1998. Above-and below-ground biomass and production by *Thalassia testudinum* in a tropical reef lagoon. *Aquatic botany* 61(1): 69-82.** Chosen because contains data about above and below ground prodcutuctivity in seagrasses.  **Rodríguez-Martínez, R. E., Ruíz-Rentería, F., Tussenbroek, B. V., Barba-Santos, G., Escalante-Mancera, E., Jordán-Garza, G., & Jordán-Dahlgren, E. 2010. Environmental state and tendencies of the Puerto Morelos CARICOMP site, Mexico. *Revista de biología tropical*5: 23-43.** Chosen because contains data about seagrass biomass.  **Gallegos, M. E., Merino, M., Marba, N., & Duarte, C. M. 1993. Biomass and dynamics of *Thalassia testudinum* in the Mexican Caribbean-elucidating rhizome growth. *Marine Ecology Progress Series* 95(1-2): 185-192.** Chosen because contains data about seagrass biomass variation.  **Enríquez, S., Marbà, N., Duarte, C. M., Van Tussenbroek, B. I., & Reyes-Zavala, G. 2001. Effects of seagrass *Thalassia testudinum* on sediment redox. *Marine Ecology Progress Series* 219: 149-158.** Chosen because contains data about carbon stocks in seagrasses soils.  **Poumian-Tapia, M. & Ibarra-Obando, S. E. 1999. Demography and biomass of the seagrass *Zostera marina* in a Mexican coastal lagoon. *Estuaries and Coasts* 22(4): 837-847.** Chosen because contains data about seagrass biomass.  **Nugent, R. S., Jordan, E. & de la Torre, R. 1978. Investigaciónes preliminares de la biomasa de *Thalassia testudinum* Konig, en la costa del Caribe Mexicano. Nota científica. *An. Centro Cienc. del Mar y Limnol*. 5(1): 247-254.** Chosen because contains data about seagrass biomass.  **Arellano-Méndez, L. U. 2011. Estructura de los pastos marinos y su relación con la variabilidad ambiental a diferentes escalas espaciales. Tesis de Doctorado. Centro de Investigación y de Estudios Avanzados del Instituto Politécnico Nacional (CINVESTAV-IPN), Unidad Mérida, Yucatán, México. 215 pp.** Chosen because contains data about seagrass biomass variation and physicochemical variables.  **Santamaría-Gallegos, N. A., Félix-Pico, E. F., Sánchez-Lizaso, J. L. & Riosmena-Rodríguez, R. 2007. Ecología de la fanerógama *Zostera marina* en el sistema lagunar Bahía Magdalena–Bahía Almejas. Estudios ecológicos en Bahía Magdalena. CICIMAR-IPN, La Paz, 101-112.** Chosen because contains data about seagrass biomass.  **Gallegos Martínez, M., Merino, M., Rodriguez, A., Marbá, N. & Duarte, C. 1994. Growth patterns and demographyof pioneer Caribbean seagrasses *Halodule wrightii* and *Syringodiun filiforme* K. *Marine Ecology Progress Series* 109: 99-104.** Chosen because contains data about seagrass biomass.  **Herrera-Silveira, J.A., Ramírez Ramírez, J., Medina Gómez, I., Valdez J., Reyes, O., Cámara, J., Caamal, J., y López-Herrera, M. 2008. Monitoreo 2008 de indicadores de salud del ecosistema de pastos marinos del Parque Nacional Costa Occidental de Isla Mujeres, Punta Cancún y Punta Nizúc. Informe Final, CINVESTAV-CONANP.** Chosen because contains data about seagrass biomass variation and physicochemical variables.  **Ruiz-Zárate, M. A., Espinoza-Avalos, J., Carricart-Ganivet, J. P. & Fragoso, D. 2000. Relationships between *Manicina areolata* (Cnidaria: Scleractinia), *Thalassia testudinum* (Anthophyta) and *Neogoniolithon sp* (Rhodophyta). *Marine Ecology Progress Series* 206: 135-146.** Chosen because contains data about seagrass biomass.  **Herrera-Silveira JA., Mendoza-Martínez J., Morales-Ojeda S., Camacho-Rico A., Medina-Gómez I., Ramírez-Ramírez I., López-Herrera M., Pech-Poot E., Pérez-Martínez O., Pech-Cárdenas M, Cota-Lucero T., Teutli-Hernández C. 2018b. Database of carbon stocks in seagrasses of Mexico. *Elementos para Políticas Públicas* 2(1): 45-52.** Chosen because contains data about above and below ground carbon stocks in seagrasses calculated with a standarized metodology.  **Cabello-Pasini, A., Muñiz-Salazar, R. & Ward, D. H. 2003. Annual variations of biomass and photosynthesis in *Zostera marina* at its southern end of distribution in the North Pacific. *Aquatic botany* 76(1): 31-47.** Chosen because contains data about seagrass biomass.  **Santa maria-Gallegos, N. 1996. Ciclo de crecimiento y fenología de la fanerógama *Zostera marina* L. en Punta Arena, Bahía Concepción, BCS Mexico. Tesis de Maestría. IPN. CICIMAR. Baja California Sur, México. 97 pp.** Chosen because contains data about seagrass biomass variation.  **Santa maria, N., Felix-Pic, E. F., Sánchez-Lizaso, J. L., and Palomares-Garcia, J. R. 1999. Temporal Coindidence of the annual seagrass *Zostera marina* and juvenile scallops *Argopecten ventricosus* (Sowerby 11, 1842) in, Bahia Concepcion, Mexico. *Journal of Shellfish Research* Vol. 18(2), 415-418.** Chosen because contains data about seagrass biomass variation.  **Meling-López, A. E., & Ibarra-Obando, S. E. 1999. Annual life cycles of two *Zostera marina* L. populations in the Gulf of California: contrasts in seasonality and reproductive effort. *Aquatic Botany* 65(1): 59-69.** Chosen because contains data about seagrass biomass variation.  **Santamarıia-Gallegos, N. A., Sánchez-Lizaso, J. L., & Félix-Pico, E. F. 2000. Phenology and growth cycle of annual subtidal seagrass in a subtropical locality. *Aquatic Botany* 66(4): 329-339.** Chosen because contains data about seagrass biomass variation.  **Cota Lucero, T. 2017. Secuestro histórico de carbono en sedimentos asociados a praderas de *Zostera marina* dentro del complejo lagunar Bahía Magdalena-Almejas. Tesis de Maestría. Universidad Autónoma de Baja California Sur. Baja California Sur, Mexico. 69 pp. *Chosen because contains data about carbon stocks in seagrasses soils.***  **Cota Lucero, T. 2014. Comparación histórica de *Zostera marina* en el complejo lagunar Bahía Magdalena-Amejas e implicaciones del cambio climático. Tesis de Licenciatura. Universidad Autónoma de Baja California Sur. Baja California Sur, México. 56 pp.** Chosen because contains data about carbon stocks in seagrasses soils.  **Terrados, J., Ramírez-García, P., Hernández-Martínez, O., Pedraza, K., & Quiroz, A. 2008. State of *Thalassia testudinum* Banks ex König meadows in the Veracruz Reef System, Veracruz, Mexico. *Aquatic botany* 88(1): 17-26.** Chosen because contains data about seagrass biomass.  **Ramírez García, P., J. Terrados M., O. Hernández Martínez, K. Pedraza, y A. Quiroz, 2007. La vegetación de *Thalassia testudinum* en los arrecifes de Hornos, Sacrificios y Enmedio: Biomasa, productividad y dinámica de crecimiento, p. 173-184. In: A. Granados Barba, L. G. Abarca Arenas y J.M. Vargas Hernández (Eds.) Investigaciones Científicas en el Sistema Arrecifal Veracruzano. Universidad Autónoma de Campeche. ISBN 968-5722-53-6. 304 p.** Chosen because contains data about seagrass biomass.  **Ibarra Morales, N., y Abarca Arenas, L.G. 2007. Distribución, abundancia y biomasa de *Thalassia testudinum* en la laguna del arrecife Sacrificios, Veracruz, p. 161-172. In: Granados Barba, A., Abarca Arenas, L.G. y Vargas Hernández, J.M. (Eds.) Investigaciones Científicas en el Sistema Arrecifal Veracruzano. Universidad Autónoma de Campeche. ISBN 968-5722-53-6. 304 p.** Chosen because contains data about seagrass biomass.  **Lot Helgueras, A., 1971. Estudios sobre fanerógamas marinas en las cercanías de Veracruz, Ver. An. Inst.Biol. UNAM Ser. Bot., 42:1-48.** Chosen because contains data about seagrass biomass  **Rivera-Guzmán, N. E., Moreno-Casasola, P., Ibarra-Obando, S. E., Sosa, V. J., and Herrera-Silveira, J. 2014. Long term state of coastal lagoons in Veracruz, Mexico: Effects of land use changes in watersheds on seagrasses habitats. Ocean & Coastal Management, 87, 30-39.** Chosen because contains data about seagrass biomass.  **Barreiro-Güemes, T., y Balderas-Cortés, J. 1991. Evaluación de algunas comunidades de productores primarios de la Laguna de la Mancha, Veracruz. *Anales del Instituto de Ciencias del Mar y Limnología* 18(2): 229-245.** Chosen because contains data about seagrass biomass.  **Rivera-Guzmán, N. E. 2008. Variación espacial y temporal de los parámetros fisicoquímicos, biológicos y de la distribución de los pastos marinos en la laguna de La Mancha. Tesis de Maestría. Instituto de Ecología, AC. Veracruz, México. 90 pp.** Chosen because contains data about seagrass biomass variation and physicochemical variables.  **Raz-Guzmán, A. & Barba, E. 2000. Seagrass biomass, distribution and associated macrofauna in southwestern Gulf of Mexico coastal lagoons. *Biol. Mar. Medit*. 7(2): 271-274.** Chosen because contains data about seagrass biomass.  **Camalich, J.M. 2001. Comparación de la Producción de *Thalassia testudinum* en Veracruz, Campeche y Yucatán. Tesis de Licenciatura. Universidad Autónoma Metropolitana de Iztapalapa, División Ciencias Biológicas de la Salud. 26 pp.** Chosen because contains data about seagrass biomass.  **Sotelo Giner, F. 2014. Distribución y biomasa del pasto marino *Halodule wrightii* en la laguna de Tampamachoco, Tuxpan Veracruz. Tesis de Maestría. Universidad Veracruzana. Facultad de Ciencias Biológicas y Agropecuarias. Veracruz, México. 54 pp.** Chosen because contains data about seagrass biomass.  **Winfield, I., Cházaro-Olvera, S. y Álvarez, F. 2007. ¿Controla la biomasa de pastos marinos la densidad de los peracáridos (Crustacea: Peracarida) en lagunas tropicales? *Revista de biología* *tropical* 55(1): 43-51.** Chosen because contains data about seagrass biomass.  **Herrera-Silveira, J.A. Arellano-Mendez, L., & Morales-Ojeda, S. 2011. Report of the Pilot Project Natural Habitat and Ecosystem Conservation of Coastal and Marine Zones of the Gulf of Mexico: Seagrass beds characterization for restoration pilot project: Phase I-2011. Integrated Assessment and Management of the Gulf of Mexico large marine ecosystem project. 37 pp.** Chosen because contains data about seagrass biomass.  **Yáñez-Arancibia, A. & Day Jr, J. W. 1982. Ecological characterization of Terminos Lagoon, a tropical lagoon-estuarine system in the southern Gulf of Mexico. *Oceanol. Acta* 5(4): 431-440.** Chosen because contains data about seagrass biomass.  **Hornelas, Y. D. J. 1975. Comparación de la biomasa, densidad y de algunos aspectos morfométricos de la fanerógama marina *Thalassia testudinum*, König, 1805 en tres diferentes áreas geográficas del Golfo de México. Tesis de Licenciatura. México, D.F. 54 pp.** Chosen because contains data about seagrass biomass.  **Moore, K. A. & Wetzel, R. L. 1988. The distribution and productivity of seagrasses in the Terminos lagoon. Ecology of Coastal Ecosystems in the southern Gulf of México-The Términos lagoon region, Universidad Nacional Autónoma de México, México, 207-220.** Chosen because contains data about seagrass biomass variation.  **Consultores en Gestión, Política y Planificación Ambiental, S.C. 2014. Proyecto Libramiento Sur Ciudad del Carmen (PLS-C). 21 pp.** Chosen because contains data about seagrass biomass.  **López-Herrera, M. 2011. Caracterización de las variables morfométricas y estructurales de *Thalassia testudinum* y su relación con la hidroquímica y sedimentos en la Bahía de Campeche frente a la ciudad de Campeche. Tesis de Licenciatura. Universidad Autónoma de Yucatán. Yucatán, México. 57 pp.** Chosen because contains data about seagrass biomass.  **Medina-Gómez, I. & Herrera-Silveira, J. A. 2006. Primary production dynamics in a pristine groundwater influenced coastal lagoon of the Yucatan Peninsula. *Continental Shelf Research* 26(8): 971-986.** Chosen because contains data about seagrass biomass.  **Herrera-Silveira, J. A. 1994. Phytoplankton productivity and submerged macrophyte biomass variation in a tropical coastal lagoon with ground water discharge. *Vie Milieu* 44(3/4): 257-266.** Chosen because contains data about seagrass biomass variation.  **Aguayo, C. M. 2004. Estructura de la vegetación acuática sumergida como bioindicador de la calidad del agua en una zona costera tropical. Tesis de Maestría. Centro de Investigación y de Estudios Avanzados del Instituto Politécnico Nacional (CINVESTAV-IPN), Unidad Mérida, Yucatán, México. 113 pp.** Chosen because contains data about seagrass biomass.  **Herrera-Silveira, J. A. 1998. Overview and characterization of the hydrology and primary producer communities of selected coastal lagoons of Yucatán, México. *Aquatic Ecosystem Health & Management* 1(3-4): 353-372.** Chosen because contains data about seagrass biomass. | 5 |
| Risk of bias within studies | 19 | **Present data on risk of bias of each study and, if available, any outcome level assessment (see item 12).**  NA | NA |
| Results of individual studies | 20 | **For all outcomes considered (benefits or harms), present, for each study: (a) simple summary data for each intervention group (b) effect estimates and confidence intervals, ideally with a forest plot**.  NA | NA |
| Synthesis of results | 21 | **Present results of each meta-analysis done, including confidence intervals and measures of consistency.**  NA | NA |
| Risk of bias across studies | 22 | **Present results of any assessment of risk of bias across studies (see Item 15).**  NA | NA |
| Additional analysis | 23 | **Give results of additional analyses, if done (e.g., sensitivity or subgroup analyses, meta-regression [see Item 16]).**  NA | NA |
| **DISCUSSION** | | |  |
| Summary of evidence | 24 | **Summarize the main findings including the strength of evidence for each main outcome; consider their relevance to key groups (e.g., healthcare providers, users, and policy makers).**  The results indicate that there is missing information at regional level, this constitute an opportunity area for researchers.  First Mexican average numbers, reported in this work for blue carbon, considering the official extension for both ecosystems in Mexico, indicate that mangrove contributions is 237.7 Tg C_org_ and seagrasses number correspond to 48.1 Tg C_org_. Those numbers constitute a baseline useful for stakeholders.  The identification of the characteristics of sites where the stocks are larger could help to identify priority sites, states or regions for restoration or protection, such as those related to humid climate, or related to the vegetation type such as peten-type mangroves (728 ± 230 Mg C_org_ ha^-1^) where the highest values for mangroves were identified and in the case of seagrasses those with low hydrodynamic of water.  The estimated emissions from the land use change under a conservative approach in mangroves of Mexico were around 24 Tg CO_2_e in the last 20 years. Therefore, the incorporation of the “blue carbon” initiative and the mechanism of carbon market into public policies is an attractive win-win opportunity which trend towards a suitable management for those ecosystems. | 6-14 |
| Limitations | 25 | **Discuss limitations at study and outcome level (e.g., risk of bias), and at review-level (e.g., incomplete retrieval of identified research, reporting bias).**  The limitations at study level were related to the availability of research or data in each locality, region, etc. This could affect the accuracy of the estimations and increase the risk of bias. Due to research papers related to blue carbon are emerging and even there are protocols, at review level we noticed they have not been implemented yet in available scientific documents at country level. Then the information included was out of standardized protocols and this constitute a source of risk of bias. No uncertainty information or dispersion values were available at review level. | 13-16 |
| Conclusions | 26 | **Provide a general interpretation of the results in the context of other evidence, and implications for future research.**  Mexico has great potential to contribute to mitigation of the effects of climate change through the conservation and restoration of blue carbon ecosystems, especially mangroves and seagrasses.  Carbon fluxes, such as the capture and exchange of methane, particulate organic carbon (POC) and dissolved organic carbon (DOC), are carried out within mangroves. The most widely used estimate of carbon capture is the measurement of ecosystem productivity, and despite the importance that has been given to the export of organic carbon between mangroves and coastal systems, arising with the theory of "outwelling" proposed by Odum & Heald, (1972), the number of quantitative estimates remains very limited.  Most of the studies in Mexico lack environmental information (interstitial salinity, soil nutrients, hydroperiod), and the increase in litterfall production during the rainy season reflects the availability of fresh water from precipitation and groundwater (Santini et al., 2015). | 16-17 |
| **FUNDING** | | |  |
| Funding | 27 | **Describe sources of funding for the systematic review and other support (e.g., supply of data); role of funders for the systematic review.**  This work was supported by United Nations Development Programme (No.CSP-2016-057) this project pay to persons who make the systematic review which were included in datasets also finance workshops and sampling in pilot sites included in dataset.  National Commission of Natural Protected Areas (Mexico) (No.FN009 & No.KN003), National Forestry Commission (Mexico) (No.137252), Ministry of Environment and Natural Resources (Mexico) (No. S0010-1108099) pay for some laboratory projects used as data source. | Supplementary information |

*From:*  Moher D, Liberati A, Tetzlaff J, Altman DG, The PRISMA Group (2009). Preferred Reporting Items for Systematic Reviews and Meta-Analyses: The PRISMA Statement. PLoS Med 6(7): e1000097. doi:10.1371/journal.pmed1000097

For more information, visit: **www.prisma-statement.org**.

Page 2 of 2
